# Local Homeostatic Regulation of the Spectral Radius of Echo-State Networks

**DOI:** 10.3389/fncom.2021.587721

**Published:** 2021-02-24

**Authors:** Fabian Schubert, Claudius Gros

**Affiliations:** Institute for Theoretical Physics, Goethe University Frankfurt am Main, Frankfurt am Main, Germany

**Keywords:** recurrent networks, homeostasis, synaptic scaling, echo-state networks, reservoir computing, spectral radius

## Abstract

Recurrent cortical networks provide reservoirs of states that are thought to play a crucial role for sequential information processing in the brain. However, classical reservoir computing requires manual adjustments of global network parameters, particularly of the spectral radius of the recurrent synaptic weight matrix. It is hence not clear if the spectral radius is accessible to biological neural networks. Using random matrix theory, we show that the spectral radius is related to local properties of the neuronal dynamics whenever the overall dynamical state is only weakly correlated. This result allows us to introduce two local homeostatic synaptic scaling mechanisms, termed flow control and variance control, that implicitly drive the spectral radius toward the desired value. For both mechanisms the spectral radius is autonomously adapted while the network receives and processes inputs under working conditions. We demonstrate the effectiveness of the two adaptation mechanisms under different external input protocols. Moreover, we evaluated the network performance after adaptation by training the network to perform a time-delayed XOR operation on binary sequences. As our main result, we found that flow control reliably regulates the spectral radius for different types of input statistics. Precise tuning is however negatively affected when interneural correlations are substantial. Furthermore, we found a consistent task performance over a wide range of input strengths/variances. Variance control did however not yield the desired spectral radii with the same precision, being less consistent across different input strengths. Given the effectiveness and remarkably simple mathematical form of flow control, we conclude that self-consistent local control of the spectral radius via an implicit adaptation scheme is an interesting and biological plausible alternative to conventional methods using set point homeostatic feedback controls of neural firing.

## 1. Introduction

Cortical networks are highly recurrent, a property that is considered to be crucial for processing and storing temporal information. For recurrent networks to remain stable and functioning, the neuronal firing activity has to be kept within a certain range by autonomously active homeostatic mechanisms. It is hence important to study homeostatic mechanisms on the level of single neurons, as well as the more theoretic question of characterizing the dynamic state that is to be attained on a global network level. It is common to roughly divide adaptation mechanisms into intrinsic homeostasis, synaptic homeostasis, and metaplasticity.

Synaptic scaling was identified as a mechanism that can postsynaptically regulate neural firing by adjusting synaptic efficacies in a proportional, multiplicative way. This finding has led to numerous studies investigating the role of synaptic scaling in controlling neural network activity (Turrigiano et al., 1998; Turrigiano and Nelson, 2000; Turrigiano, 2008) and in stabilizing other plasticity mechanisms (van Rossum et al., 2000; Stellwagen and Malenka, 2006; Tetzlaff, 2011; Toyoizumi et al., 2014). Indeed, synaptic scaling has proven successful in stabilizing activity in recurrent neural networks (Lazar et al., 2009; Remme and Wadman, 2012; Zenke et al., 2013; Effenberger and Jost, 2015; Miner and Triesch, 2016). However, these studies either used synaptic scaling as the sole homeostatic mechanism (Remme and Wadman, 2012; Zenke et al., 2013) or resorted to a variant of synaptic scaling where the scaling is not dynamically determined through a control loop using a particular target activity, but rather by a fixed multiplicative normalization rule (Lazar et al., 2009; Effenberger and Jost, 2015; Miner and Triesch, 2016). Therefore, these homeostatic models cannot account for higher moments of temporal activity patterns, i.e., their variance, as this would require at least the tuning of two parameters (Cannon and Miller, 2017).

Within more abstract models of rate encoding neurons, intrinsic homeostasis and synaptic scaling essentially correspond to adjusting a bias and gain factor on the input entering a nonlinear transfer function. Within this framework, multiple dual-homeostatic adaptation rules have been investigated concerning their effect on network performance. In this framework, the adaptation of the bias acts as an intrinsic plasticity mechanism for the control of the internal excitability of a neuron (Franklin et al., 1992; Abbott and LeMasson, 1993; Borde et al., 1995), while the gain factors functionally correspond to a synaptic scaling of the recurrent weights. Learning rules for these types of models were usually derived by defining a target output distribution that each neuron attempts to reproduce by changing neural gains and biases (Steil, 2007; Triesch, 2007; Schrauwen et al., 2008; Boedecker et al., 2009), or were directly derived from an information-theoretic measure (Bell and Sejnowski, 1995).

While these studies did indeed show performance improvements by optimizing local information transmission measures, apparently, optimal performance can effectively be traced back to a global parameter, the spectral radius of the recurrent weight matrix (Schrauwen et al., 2008). Interestingly, to our knowledge, theoretical studies on spiking neural networks did not explicitly consider the spectral radius as a parameter affecting network dynamics. Still, the theory of balanced states in spiking recurrent networks established the idea that synaptic strengths should scale with $1/\sqrt{k}$, where *k* is the average number of afferent connections (Van Vreeswijk and Sompolinsky, 1998). According to the circular law of random matrix theory, this scaling rule simply implies that the spectral radius of the recurrent weight matrix remains finite as the number of neurons *N* increases. More recent experiments on cortical cultures confirm this scaling (Barral and D'Reyes, 2016).

In the present study, we investigated whether the spectral radius of the weight matrix in a random recurrent network can be regulated by a combination of intrinsic homeostasis and synaptic scaling. Following the standard echo-state framework, we used rate encoding tanh-neurons as the model of choice. However, aside from their applications as efficient machine learning algorithms, echo state networks are potentially relevant as models of information processing in the brain (Nikolić et al., 2009; Hinaut et al., 2015; Enel et al., 2016). Note in this context that extensions to layered ESN architectures have been presented by Gallicchio and Micheli (2017), which bears a somewhat greater resemblance to the hierarchical structure of cortical networks than the usual shallow ESN architecture. This line of research illustrates the importance of examining whether local and biological plausible principles exist that would allow to tune the properties of the neural reservoir to the “edge of chaos” (Livi et al., 2018), particularly when a continuous stream of inputs is present. The rule has to be independent of both the network topology, which is not locally accessible information, and the distribution of synaptic weights.

We propose and compare two unsupervised homeostatic mechanisms, which we denote by flow control and variance control. Both regulate the mean and variance of neuronal firing such that the network works in an optimal regime concerning sequence learning tasks. The mechanisms act on two sets of node-specific parameters, the biases *b*_*i*_, and the neural gain factors *a*_*i*_. We restricted ourselves to biologically plausible adaptation mechanisms, viz adaptation rules for which the dynamics of all variables are local, i.e., bound to a specific neuron. Additional variables enter only when locally accessible. In a strict sense, this implies that local dynamics are determined exclusively by the neuron's dynamical variables and by information about the activity of afferent neurons. Being less restrictive, one could claim that it should also be possible to access aggregate or mean-field quantities that average a variable of interest over the population. For example, nitric oxide is a diffusive neurotransmitter that can act as a measure for the population average of neural firing rates (Sweeney et al., 2015).

Following a general description of the network model, we introduce both adaptation rules and evaluate their effectiveness in tuning the spectral radius in sections 2.4 and 2.5. We assess the performance of networks that were subject to adaptation in section 2.7, using a nonlinear sequential memory task. Finally, we discuss the influence of node-to-node cross-correlations within the population in section 2.8.

## 2. Results

### 2.1. Model

A full description of the network model and parameters can be found in the methods section. We briefly introduce the network dynamics as

(1) $$\begin{array}{ll} x_{i}\left(t\right) & =x_{\text{r},i}\left(t\right)+I_{i}\left(t\right) \end{array}$$

(2) $$x_{\text{r},i}(t):=a_{i}\sum_{j=1}^{N}W_{ij}y_{j}(t-1)$$

(3) $$y_{i}(t)=\tanh\left(x_{i}(t)-b_{i}\right)\text{​}.$$

Each neuron's membrane potential *x*_*i*_ consists of a recurrent contribution *x*_r, *i*_(*t*) and an external input *I*_*i*_(*t*). The biases *b*_*i*_ are subject to the following homeostatic adaptation:

(4) $$\begin{array}{l} b_{i}\left(t\right)=b_{i}\left(t-1\right)+\epsilon_{\text{b}}\left[y_{i}\left(t\right)-\mu_{\text{t}}\right]. \end{array}$$

Here, μ_t_ defines a target for the average activity and ϵ_b_ is the adaptation rate.

The local parameters *a*_*i*_ act as scaling factors on the recurrent weights. We considered two different forms of update rules. Loosely speaking, both drive the network toward a certain dynamical state which corresponds to the desired spectral radius. The difference between them lies in the variables characterizing this state: While flow control defines a relation between the variance of neural activity and the variance of the total recurrent synaptic current, variance control does so by a more complex relation between the variance of neural activity and the variance of the synaptic current from the external input.

#### 2.1.1. Flow Control

The first adaptation rule, flow control, is given by

(5) $$a_{i}(t)=a_{i}(t-1)[1+\epsilon_{\text{a}}\Delta R_{i}(t)],\;\Delta R_{i}(t)=R_{\text{t}}^{2}y_{i}^{2}(t-1)-x_{\text{r},i}^{2}(t)\text{ ​}.$$

The parameter *R*_t_ is the desired target spectral radius and ϵ_a_ the adaptation rate of the scaling factor. The dynamical variables $y_{i}^{2}$ and $x_{\text{r},i}^{2}$ have been defined before in Equations (1) and (2). We also considered an alternative global update rule where Δ*R*_*i*_(*t*) is given by

(6) $$\Delta R_{i}(t)=\frac{1}{N}\left[R_{\text{t}}^{2}||y\left(t-1\right){||}^{2}-||x_{\text{r}}(t){||}^{2}\right],$$

where || · || denotes the Euclidean vector norm. However, since this is a non-local rule, it only served as a comparative model to Equation (5) when we investigated the effectiveness of the adaptation mechanism. Three key assumptions enter flow control, Equation (5):

- Represented by *x*_r, *i*_(*t*), we assume that there is a physical separation between the recurrent input that a neuron receives and its external inputs. This is necessary because *x*_r, *i*_(*t*) is explicitly used in the update rule of the synaptic scaling factors.
- Synaptic scaling only affects the weights of recurrent connections. However, this assumption is not crucial for the effectiveness of our plasticity rule, as we were mostly concerned with achieving a preset spectral radius for the recurrent weight matrix. If instead the scaling factors acted on both the recurrent and external inputs, this would lead to an “effective” external input $I_{i}^{\prime }\left(t\right):=a_{i}I_{i}\left(t\right)$. However, *a*_*i*_ only affecting the recurrent input facilitated the parameterization of the external input by means of its variance (see section 2.7), a choice of convenience.
- For (5) to function, neurons need to able to represent and store squared neural activities.

Whether these three preconditions are satisfied by biological neurons needs to be addressed in future studies.

#### 2.1.2. Variance Control

The second adaptation rule, variance control, has the form

(7) $$a_{i}(t)=a_{i}(t-1)+\epsilon_{\text{a}}\left[\sigma_{\text{t},i}^{2}(t)-{\left(y_{i}(t)-\mu_{i}^{\text{y}}(t)\right)}^{2}\right]$$

(8) $$\sigma_{\text{t},i}^{2}(t)\;=1-\frac{1}{\sqrt{1+2R_{\text{t}}^{2}y_{i}{\left(t\right)}^{2}+2\sigma_{\text{ext},i}^{2}(t)}}.$$

Equation (7) drives the average variance of each neuron toward a desired target variance $\sigma_{\text{t},i}^{2}\left(t\right)$ at an adaptation rate ϵ_a_ by calculating the momentary squared difference between the local activity *y*_*i*_(*t*) and its trailing average $\mu_{i}^{\text{y}}\left(t\right)$. Equation (8) calculates the target variance as a function of the target spectral radius *R*_t_, the current local square activity $y_{i}^{2}\left(t\right)$ and a trailing average $\sigma_{\text{ext},i}^{2}\left(t\right)$ of the local variance of the external input signal. When all *a*_*i*_(*t*) reach a steady state, the average neural variance equals the target given by (8). According to a mean-field approach that is described in section 5.6, reaching this state then results in a spectral radius *R*_a_ that is equal to the target *R*_t_ entering (8). Intuitively, it is to be expected that $\sigma_{\text{t},i}^{2}$ is a function of both the spectral radius and the external driving variance: The amount of fluctuations in the network activity is determined by the dynamic interplay between the strength of the external input as well as the recurrent coupling.

A full description of the auxiliary equations and variables used to calculate $\mu_{i}^{\text{y}}\left(t\right)$ and $\sigma_{\text{ext},i}^{2}\left(t\right)$ can be found in section 5.1.

Similar to flow control, we also considered a non-local version for comparative reasons, where (8) is replaced with

(9) $$\sigma_{\text{t},i}^{2}(t)=1-\frac{1}{\sqrt{1+2R_{\text{t}}^{2}||y(t)\text{|}{\text{|}}^{2}/N+2\sigma_{\text{ext},i}^{2}(t)}}\text{ ​}.$$

Again, ||·|| denotes the Euclidean norm. Before proceeding to the results, we discuss the mathematical background of the proposed adaptation rules in some detail.

### 2.2. Autonomous Spectral Radius Regulation

There are some interesting aspects to the theoretical framework at the basis of the here proposed regulatory scaling mechanisms.

The circular law of random matrix theory states that the eigenvalues λ_*j*_ are distributed uniformly on the complex unit disc if the elements of a real *N* × *N* matrix are drawn from distributions having zero mean and standard deviation $1/\sqrt{N}$ (Tao and Vu, 2008). Given that the internal weight matrix $\hat{W}$ ($\overset{\wedge }{\cdot }$ denoting matrices) with entries *W*_*ij*_ has *p*_r_*N* non-zero elements per row (*p*_r_ is the connection probability), the circular law implies that the spectral radius of *a*_*i*_*W*_*ij*_, the maximum of |λ_*j*_|, is unity when the synaptic scaling factors *a*_*i*_ are set uniformly to 1/σ_w_, where σ_w_ is the standard deviation of *W*_*ij*_. Our goal is to investigate adaptation rules for the synaptic scaling factors that are based on dynamic quantities, which includes the membrane potential *x*_*i*_, the neural activity *y*_*i*_ and the input *I*_*i*_.

The circular law, i.e., a *N* × *N* matrix with i.i.d. entries with zero mean and 1/*N* variance approaching a spectral radius of one as *N* → ∞, can be generalized. Rajan and Abbott (2006) investigated the case where the statistics of the columns of the matrix differ in their means and variances: given row-wise E-I balance for the recurrent weights, the square of the spectral radius of a random *N* × *N* matrix whose columns have variances $\sigma_{i}^{2}$ is $N{\langle \sigma_{i}^{2}\rangle }_{i}$ for *N* → ∞. Since the eigenvalues are invariant under transposition, this result also holds for row-wise variations of variances and column-wise E-I balance. While the latter is not explicitly enforced in our case, deviations from this balance are expected to tend to zero for large *N* given the statistical assumptions that we made about the matrix elements *W*_*ij*_. Therefore, the result can be applied to our model, where node-specific gain factors *a*_*i*_ are applied to each row of the recurrent weight matrix. Thus, the spectral radius *R*_a_ of the *effective random matrix* ${\hat{W}}_{\text{a}}$ with entries *a*_*i*_*W*_*ij*_ [as entering (2)] is

(10) $$\begin{array}{l} R_{\text{a}}^{2}≊\frac{1}{N}\underset{i}{\sum }R_{\text{a},i}^{2},\;R_{\text{a},i}^{2}:=a_{i}^{2}\underset{j}{\sum }{\left(W_{ij}\right)}^{2}, \end{array}$$

for large *N*, when assuming that the distribution underlying the *bare weight matrix* $\hat{W}$ with entries *W*_*ij*_ has zero mean. Note that $R_{\text{a}}^{2}$ can be expressed alternatively in terms of the Frobenius norm ${\left\|{\hat{W}}_{\text{a}}\right\|}_{\text{F}}$, via

(11) $$R_{\text{a}}^{2}≊{\left\|{\hat{W}}_{\text{a}}\right\|}_{\text{F}}^{2}/N.$$

We numerically tested Equation (10) for *N* = 500 and heterogeneous random sets of *a*_*i*_ drawn from a uniform [0, 1]-distribution and found a very close match to the actual spectral radii (1–2% relative error). Given that the *R*_a, *i*_ can be interpreted as per-site estimates for the spectral radius, one can use the generalized circular law (10) to regulate *R*_a_ on the basis of local adaptation rules, one for every *a*_*i*_.

For the case of flow control, the adaptation rule is derived using a comparison between the variance of neural activity that is present in the network with the recurrent contribution to the membrane potential. A detailed explanation is presented in sections 2.6, 5.5. In short, we propose that

(12) $${\langle ∥x_{\text{r}}(t){∥}^{2}\rangle }_{\text{t}}≊R_{\text{a}}^{2}{\langle ∥y(t-1){∥}^{2}\text{ 〉}}_{\text{t}},$$

where *x*_r, *i*_ is the recurrent contribution to the membrane potential *x*_*i*_. This stationarity condition leads to the adaptation rule given in Equation (5). An analysis of the dynamics of this adaptation mechanisms can be found in section 5.5.

Instead of directly imposing Equation (12) via an appropriate adaptation mechanism, we also considered the possibility of transferring this condition into a set point for the variance of neural activities as a function the external driving. To do so, we used a mean-field approach to describe the effect of recurrent input onto the resulting neural activity variance. An in-depth discussion is given in section 5.6. This led to the update rule given by Equations (7) and (8) for variance control.

### 2.3. Testing Protocols

We used several types of input protocols for testing the here proposed adaptation mechanisms, as well as for assessing the task performance discussed in section 2.7. The first two variants concern distinct biological scenarios:

- *Binary*. Binary input sequences correspond to a situation when a neural ensemble receives input dominantly from a singular source, which itself has only two states, being either active or inactive. Using binary input sequences during learning is furthermore consistent with the non-linear performance test considered here for the echo-state network as a whole, the delayed XOR-task. See section 2.7. For symmetric binary inputs, as used, the source signal *u*(*t*) is drawn from ±1.
- *Gaussian*. Alternatively one can consider the situation that a large number of essentially uncorrelated input streams are integrated. This implies random Gaussian inputs signals. Neurons receive in this case zero-mean independent Gaussian noise.

Another categorical dimension concerns the distribution of the afferent synaptic weights. Do all neurons receive inputs with the same strength, or not? As a quantifier for the individual external input strengths, the variances $\sigma_{\text{ext},i}^{2}$ of the local external input currents where taken into account. We distinguished two cases

- *Heterogeneous*. In the first case, the $\sigma_{\text{ext},i}^{2}$ are quenched random variables. This means that each neuron is assigned a random value $\sigma_{\text{ext},i}^{2}$ before the start of the simulation, as drawn from a half-normal distribution parameterized by σ = σ_ext_. This ensures that the expected average variance $\langle \sigma_{\text{ext},i}^{2}\rangle$ is given by $\sigma_{\text{ext}}^{2}$.
- *Homogeneous*. In the second case, all $\sigma_{\text{ext},i}^{2}$ are assigned the identical global value $\sigma_{\text{ext}}^{2}$.

Overall, pairing “binary” vs. “Gaussian” and “heterogeneous” vs. “homogeneous,” leads to a total of four different input protocols, i.e., “heterogeneous binary,” “homogeneous binary,” “heterogeneous Gaussian,” and “homogeneous Gaussian.” If not otherwise stated, numerical simulations were done using networks with *N* = 500 sites and a connection probability *p*_r_ = 0.1.

### 2.4. Performance Testing of Flow Control

**Figure 1:**
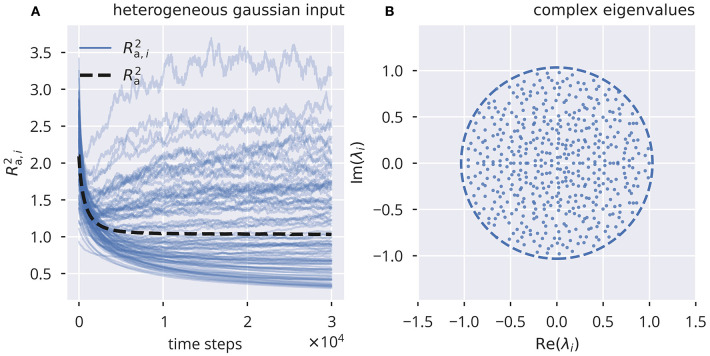
Online spectral radius regulation using flow control. The spectral radius *R*_a_ and the respective local estimates *R*_a, *i*_ as defined by (10). For the input protocol see section 5.3. **(A)** Dynamics of $R_{\text{a},i}^{2}$ and $R_{\text{a}}^{2}$, in the presence of heterogeneous independent Gaussian inputs. Local adaptation. **(B)** Distribution of eigenvalues of the corresponding effective synaptic matrix ${\hat{W}}_{\text{a}}$, after adaptation. The circle denotes the spectral radius.

In Figure 1, we present a simulation using flow control for heterogeneous Gaussian input with an adaptation rate $\epsilon_{\text{a}}=10^{-3}$. The standard deviation of the external driving was set to σ_ext_ = 0.5. The spectral radius of *R*_a_ of ${\hat{W}}_{\text{a}}$ was tuned to the target *R*_t_ = 1 with high precision, even though the local, row-wise estimates *R*_a, *i*_ showed substantial deviations from the target.

We further tested the adaptation with other input protocols (see section 2.3 and Supplementary Figure 1). We found that flow control robustly led to the desired spectral radius *R*_t_ under all Gaussian input protocols, while binary input caused *R*_a_ to converge to higher values than *R*_t_. However, when using global adaptation, as given by Equation (6), all input protocols resulted in the correctly tuned spectral radius (see Supplementary Figure 2).

Numerically, we found that the time needed to converge to the stationary states depended substantially on *R*_t_, slowing down when the spectral radius becomes small. It is then advantageous, as we have done, to scale the adaptation rate ϵ_a_ inversely with the trailing average ${\bar{x}}_{\text{r}}^{2}$ of $||x_{\text{r}}{||}^{2}$, viz as $\epsilon_{\text{a}}\to \epsilon_{\text{a}}/{\bar{x}}_{\text{r}}^{2}$. An exemplary plot showing the effect of this scaling is shown in **Figure 7** (see section 5.2 for further details).

To evaluate the amount of deviation from the target spectral radius under different input strengths and protocols, we plotted the difference between the resulting spectral radius and the target spectral radius for a range of external input strength, quantified by their standard deviation σ_ext_. Results for different input protocols are shown in Supplementary Figure 5. For correlated binary input, increasing the input strength resulted in stronger deviations from the target spectral radius. On the other hand, uncorrelated Gaussian inputs resulted in perfect alignment for the entire range of input strengths that we tested.

### 2.5. Performance Testing of Variance Control

**Figure 2:**
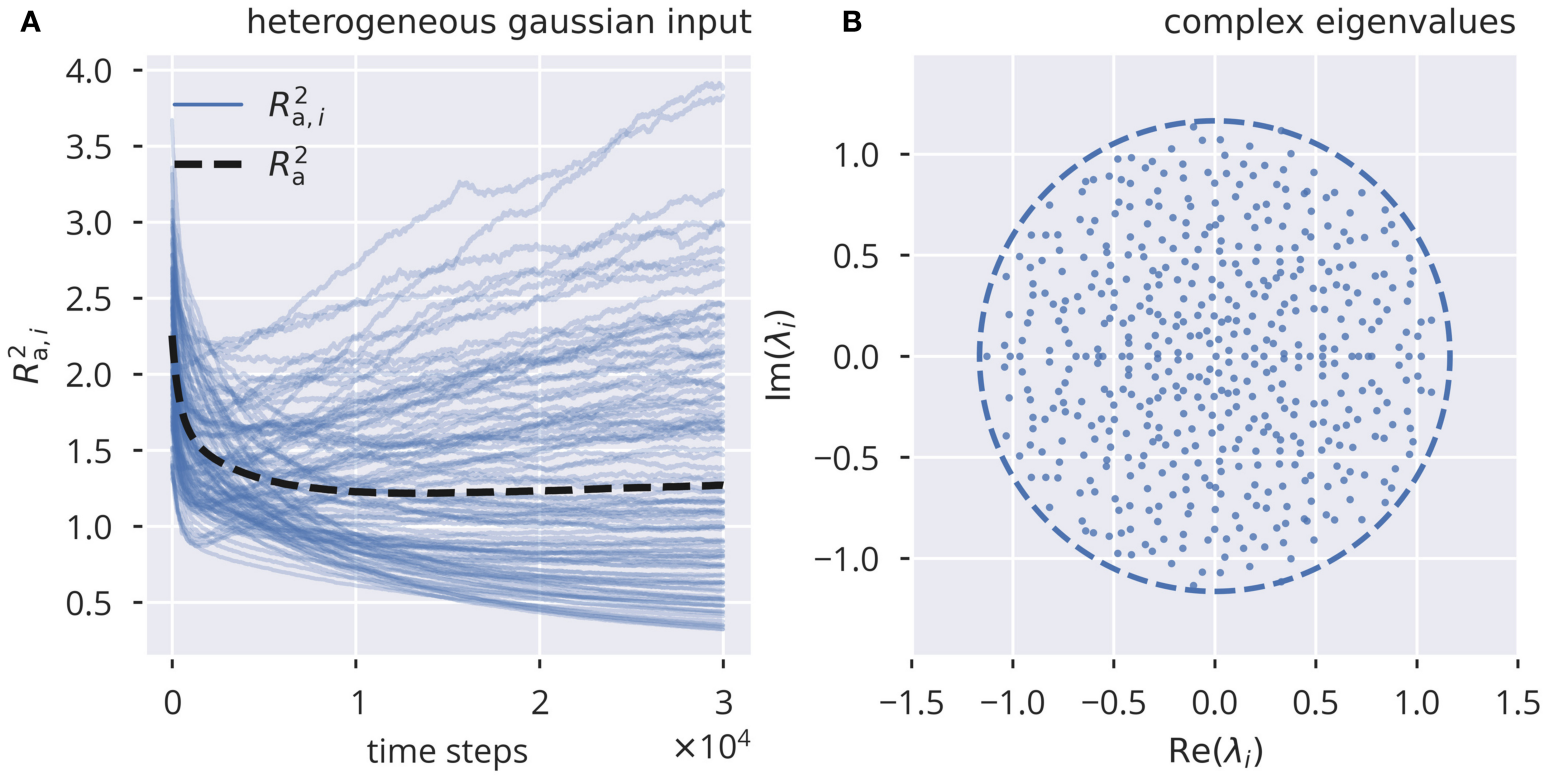
Online spectral radius regulation using variance control. The spectral radius *R*_a_ and the respective local estimates *R*_a, *i*_ as defined by (10). For the input protocol see section 5.3. **(A)** Dynamics of $R_{\text{a},i}^{2}$ and $R_{\text{a}}^{2}$, in the presence of heterogeneous independent Gaussian inputs. Local adaptation. **(B)** Distribution of eigenvalues of the corresponding effective synaptic matrix ${\hat{W}}_{\text{a}}$. The circles denote the respective spectral radius.

In comparison, variance control, shown in Figure 2, Supplementary Figure 3, resulted in notable deviations from *R*_t_, for both uncorrelated Gaussian and correlated binary input. As for flow control, we also calculated the deviations from *R*_t_ as a function of σ_ext_ (see Supplementary Figure 6). For heterogeneous binary input, deviations from the target spectral radius did not increase monotonically as a function of the input strength (Supplementary Figure 6A), reaching a peak at σ_ext_ ≈ 0.4 for target spectral radii larger than 1. For homogeneous binary input, we observed a substantial negative mismatch of the spectral radius for strong external inputs (see Supplementary Figure 6C).

Overall, we found that variance control did not exhibit the same level of consistency in tuning the system toward a desired spectral radius, even though it did perform better in some particular cases (compare Supplementary Figure 5A for large σ_ext_ with Supplementary Figure 6). Moreover, variance control exhibited deviations from the target (shown in Supplementary Figure 4) even when a global adaptation rule was used, as defined in (9). This is in contrast to the global variant of flow control, which, as stated in the previous section, robustly tuned the spectral radius to the desired value even in the presence of strongly correlated inputs.

### 2.6. Spectral Radius, Singular Values, and Global Lyapunov Exponents

Apart from the spectral radius *R*_a_ of the matrix ${\hat{W}}_{\text{a}}$, one may consider the relation between the adaptation dynamics and the respective singular values σ_*i*_ of ${\hat{W}}_{\text{a}}$. We recall that the spectrum of ${\hat{U}}_{\text{a}}={\hat{W}}_{\text{a}}^{†}{\hat{W}}_{\text{a}}$ is given by the squared singular values, $\sigma_{i}^{2}$, and that the relation $||{\text{x}}_{\text{r}}{||}^{2}={\text{y}}^{†}{\hat{W}}_{\text{a}}^{†}{\hat{W}}_{\text{a}}\text{y}$ holds. Now, assume that the time-averaged projection of neural activity **y** = **y**(*t*) onto all eigenvectors of *Û*_a_ is approximately the same, that is, there is no preferred direction of neural activity in phase space. From this idealized case, it follows that the time average of the recurrent contribution to the membrane potential can be expressed with

(13) $${\langle ∥{\text{x}}_{\text{r}}{∥}^{2}\rangle }_{\text{t}}\approx \frac{{\langle ∥\text{y}{∥}^{2}\rangle }_{\text{t}}}{N}\sum_{i}\sigma_{i}^{2}=\frac{{\langle ∥\text{y}{∥}^{2}\rangle }_{\text{t}}}{N}\sum_{i,j}{\left(a_{i}W_{ij}\right)}^{2}$$

as the rescaled average of the $\sigma_{i}^{2}$. For the second equation, we used the fact that the $\underset{i}{\sum }\sigma_{i}^{2}$ equals the sum of all matrix elements squared (Sengupta and Mitra, 1999; Shen, 2001). With (10), one finds that (13) is equivalent to ${\langle ||x_{\text{r}}{||}^{2}\rangle }_{\text{t}}=R_{a}^{2}{\langle ||y{||}^{2}\rangle }_{\text{t}}$ and hence to the flow condition (12). This result can be generalized, as done in section 5.5, to the case that the neural activities have inhomogeneous variances, while still being uncorrelated with zero mean. We have thus shown that the stationarity condition leads to a spectral radius of (approximately) unity.

It is worthwhile to note that the singular values of ${\hat{U}}_{\text{a}}={\hat{W}}_{\text{a}}^{†}{\hat{W}}_{\text{a}}$ do exceed unity when *R*_a_ = 1. More precisely, for a random matrix with i.i.d. entries, one finds in the limit of large *N* that the largest singular value is given by σ_max_ = 2*R*_a_, in accordance with the Marchenko-Pastur law for random matrices (Marčenko and Pastur, 1967). Consequently, directions in phase space exist in which the norm of the phase space vector is elongated by factors greater than one. Still, this does not contradict the fact that a unit spectral radius coincides with the transition to chaos for the non-driven case. The reason is that the global Lyapunov exponents are given by

(14) $$\begin{array}{l} \underset{n\to \infty }{\lim}\frac{1}{2n}\ln\left({\left({\hat{W}}_{\text{a}}^{n}\right)}^{†}{\hat{W}}_{\text{a}}^{n}\right) \end{array}$$

which eventually converge to ln ∥λ_*i*_∥, see Supplementary Figure 7 and Wernecke et al. (2019), where λ_*i*_ is the *i*th eigenvalue of ${\hat{W}}_{\text{a}}$. The largest singular value of the *n*th power of a random matrix with a spectral radius *R*_a_ scales like $R_{\text{a}}^{n}$ in the limit of large powers *n*. The global Lyapunov exponent goes to zero as a consequence when *R*_a_ → 1.

### 2.7. XOR-Memory Recall

To this point, we presented results regarding the effectiveness of the introduced adaptation rules. However, we did not account for their effects onto a given learning task. Therefore, we tested the performance of locally adapted networks under the delayed XOR task, which evaluates the memory capacity of the echo state network in combination with a non-linear operation. For the task, the XOR operation is to be taken with respect to a delayed pair of two consecutive binary inputs signals, *u*(*t*−τ) and *u*(*t*−τ−1), where τ is a fixed time delay. The readout layer is given by a single unit, which has the task of reproducing

(15) $$\begin{array}{l} f_{\tau }\left(t\right)=\text{XOR}\left[u\left(t-\tau \right),u\left(t-\tau -1\right)\right],\;t,\tau =1,2,..., \end{array}$$

where XOR[*u, u*′] is 0/1 if *u* and *u*′ are identical/not identical.

The readout vector **w**_out_ is trained with respect to the mean squared output error,

(16) $$\begin{array}{l} {\left||\hat{Y}{\text{w}}_{\text{out}}-{\text{f}}_{\tau }|\right|}^{2}+\alpha {\left||{\text{w}}_{\text{out}}|\right|}^{2}, \end{array}$$

using ridge regression on a sample batch of *T*_batch_ = 10*N* time steps, here for *N* = 500, and a regularization factor of α = 0.01. The batch matrix $\hat{Y}$, of size *T*_batch_ × (*N* + 1), holds the neural activities as well as one node with constant activity serving as a potential bias. Similarly, the readout (column) vector **w**_out_ is of size (*N* + 1). The *T*_batch_ entries of **f**_τ_ are the *f*_τ_ (*t*), viz the target values of the XOR problem. Minimizing (16) leads to

(17) $$\begin{array}{l} {\text{w}}_{\text{out}}={\left({\hat{Y}}^{†}\hat{Y}+\alpha^{2}\hat{\text{𝟙}}\right)}^{-1}{\hat{Y}}^{†}{\text{f}}_{\tau }. \end{array}$$

The learning procedure was repeated independently for each time delay τ. We quantified the performance by the total memory capacity, MC_XOR_, as

(18) $$\begin{array}{l} \text{M}{\text{C}}_{\text{XOR}}=\overset{\infty }{\underset{k=1}{\sum }}\text{M}{\text{C}}_{\text{XOR},k} \end{array}$$

(19) $$\begin{array}{l} \text{M}{\text{C}}_{\text{XOR},k}=\frac{\text{Co}{\text{v}}^{2}{\left[f_{k}\left(t\right),y_{\text{out}}\left(t\right)\right]}_{t}}{\text{Var}{\left[f_{k}\left(t\right)\right]}_{t}\text{Var}{\left[y_{\text{out}}\left(t\right)\right]}_{t}}. \end{array}$$

This is a simple extension of the usual definition of short term memory in the echo state literature (Jaeger, 2002). The activity $y_{\text{out}}=\overset{N+1}{\underset{i=1}{\sum }}w_{\text{out},i}y_{i}$ of the readout unit is compared in (19) with the XOR prediction task, with the additional neuron, *y*_*N*+1_ = 1, corresponding to the bias of the readout unit. Depending on the mean level of the target signal, this offset might actually be unnecessary. However, since it is a standard practice to use an intercept variable in linear regression models, we decided to include it into the readout variable *y*_out_. The variance and covariance are calculated with respect to the batch size *T*_batch_.

**Figure 3:**
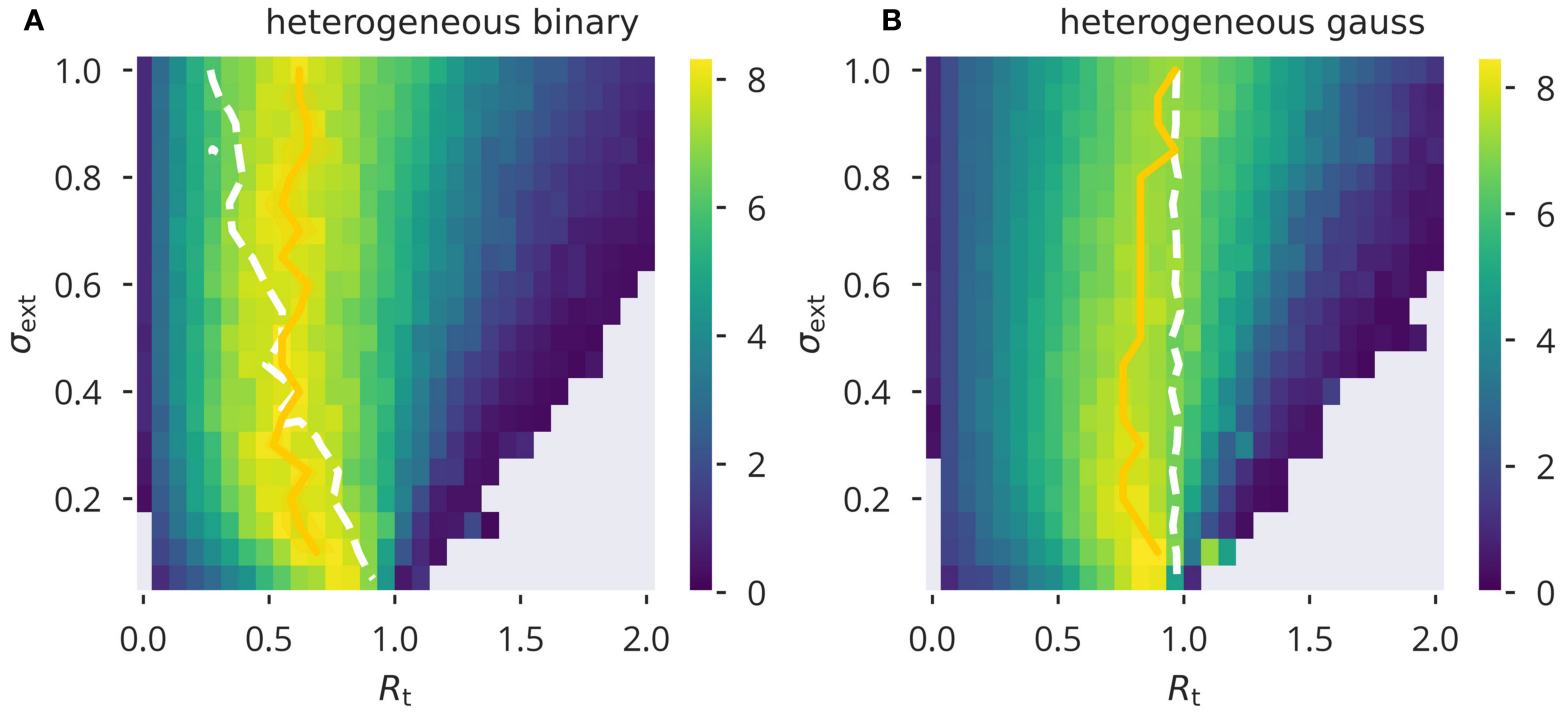
XOR performance for flow control. Color-coded performance sweeps for the XOR-performance (18) after adaptation using flow control. Averaged over five trials. The input has variance $\sigma_{\text{ext}}^{2}$ and the target for the spectral radius is *R*_t_. **(A,B)** Heterogeneous binary/Gaussian input protocols. Optimal performance for a given σ_ext_ was estimated as a trial average (yellow solid line) and found to be generally close to criticality, *R*_a_ = 1, as measured (white dashed lines).

The results for flow control presented in Figure 3 correspond to two input protocols, heterogeneous Gaussian and binary inputs. Shown are sweeps over a range of σ_ext_ and *R*_t_. The update rule (5) was applied to the network for each pair of parameters until the *a*_*i*_ values converged to a stable configuration. We then measured the task performance as described above. Note that in the case of Gaussian input, this protocol was only used during the adaptation phases. Due to the nature of the XOR task, binary inputs with the corresponding variances are to be used during performance testing. See Supplementary Figure 8 for a performance sweep using the homogeneous binary and Gaussian input protocol. Optimal performance was generally attained around the *R*_a_ ≈ 1 line. A spectral radius *R*_a_ slightly smaller than unity was optimal when using Gaussian input, but not for binary input signals. In this case the measured spectral radius *R*_a_ deviated linearly from the target *R*_t_, with increasing strength of the input, as parameterized by the standard deviation σ_ext_. Still, the locus of optimal performance was essentially independent of the input strength, with maximal performance attained roughly at *R*_t_ ≈ 0.55. Note that the line *R*_a_ = 1 joins *R*_t_ = 1 in the limit σ_ext_ → 0.

**Figure 4:**
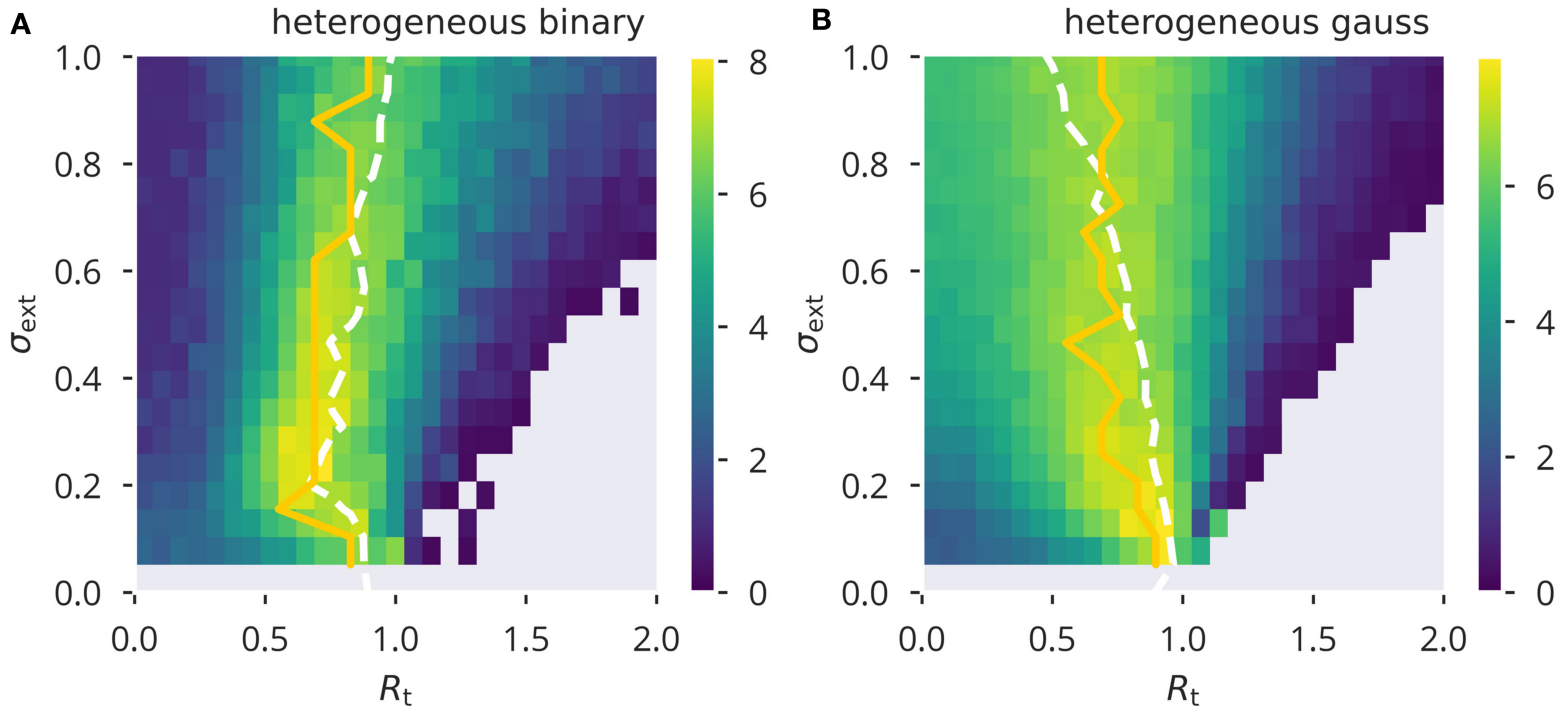
XOR performance for variance control. Color-coded performance sweeps for the XOR-performance (18) after adaptation using variance control. Averaged over five trials. The input has variance $\sigma_{\text{ext}}^{2}$ and the target for the spectral radius *R*_t_. **(A,B)** Heterogeneous binary/Gaussian input protocols. Optimal performance (yellow solid line) is in general close to criticality, *R*_a_ = 1, as measured (white dashed lines).

Comparing these results to variance control, as shown in Figure 4, we found that variance control led to an overall lower performance. To our surprise, for external input with a large variance, Gaussian input caused stronger deviations from the desired spectral radius as compared to binary input. Therefore, in a sense, it appeared to behave opposite to what we found for flow control. However, similar to flow control, the value of *R*_t_ giving optimal performance under a given σ_ext_ remained relatively stable over the range of external input strength measured. On the other hand, using homogeneous input (see Supplementary Figure 9), did cause substantial deviations from the target spectral radius when using binary input.

### 2.8. Input Induced Correlations

**Figure 5:**
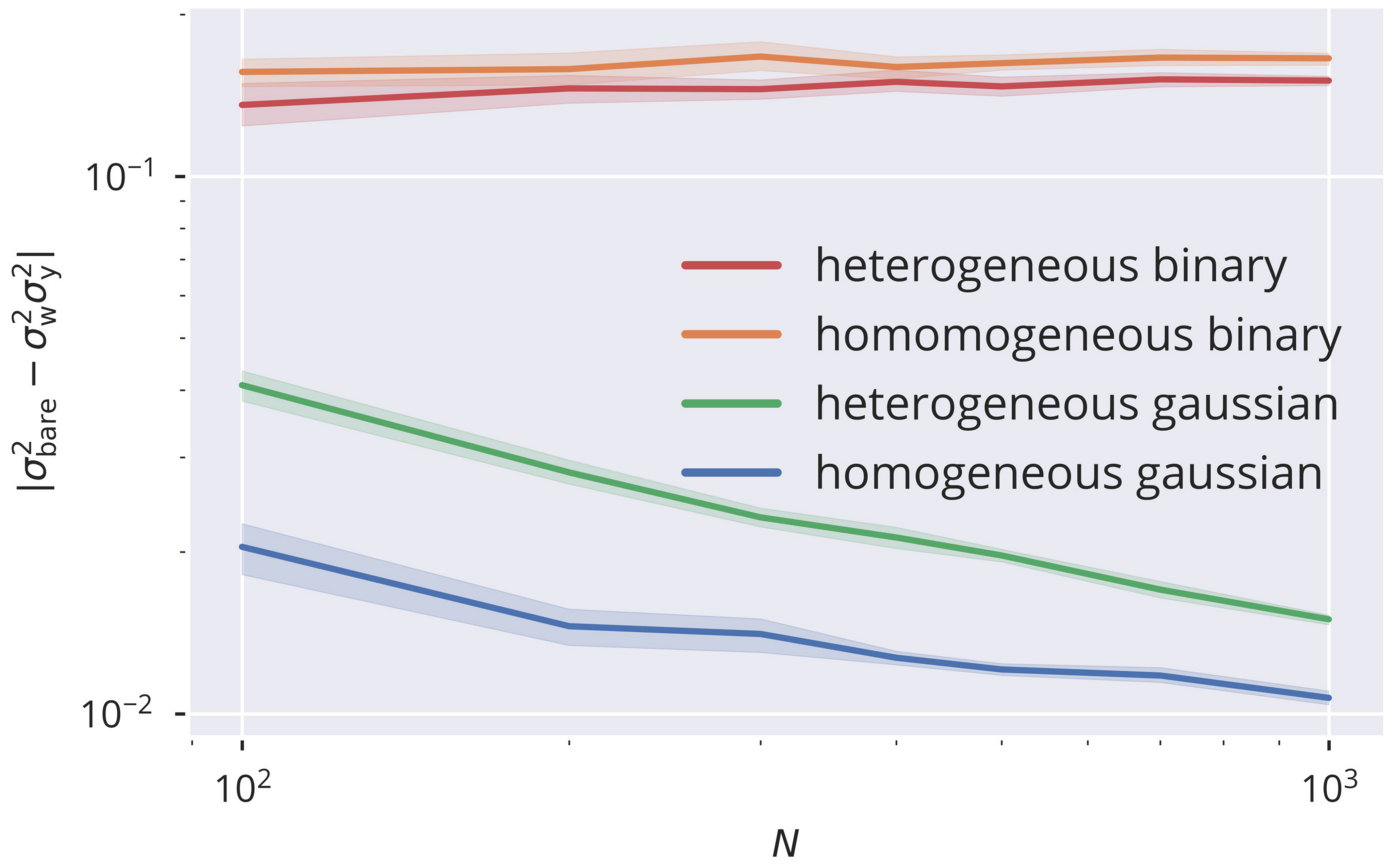
Size dependence of correlation. Comparison between the variance $\sigma_{\text{bare}}^{2}$ of the bare recurrent input $x_{\text{bare}}=\underset{j}{\sum }W_{ij}y_{j}$ with $\sigma_{\text{w}}^{2}\sigma_{\text{y}}^{2}$. Equality is given when the presynaptic activities are statistically independent. This can be observed in the limit of large network sizes *N* for uncorrelated input data streams (homogeneous and heterogeneous Gaussian input protocols), but not for correlated inputs (homogeneous and heterogeneous binary input protocols). Compare section 5.3 for the input protocols. Parameters are σ_ext_ = 0.5, *R*_a_ = 1, and μ_*t*_ = 0.05.

**Figure 6:**
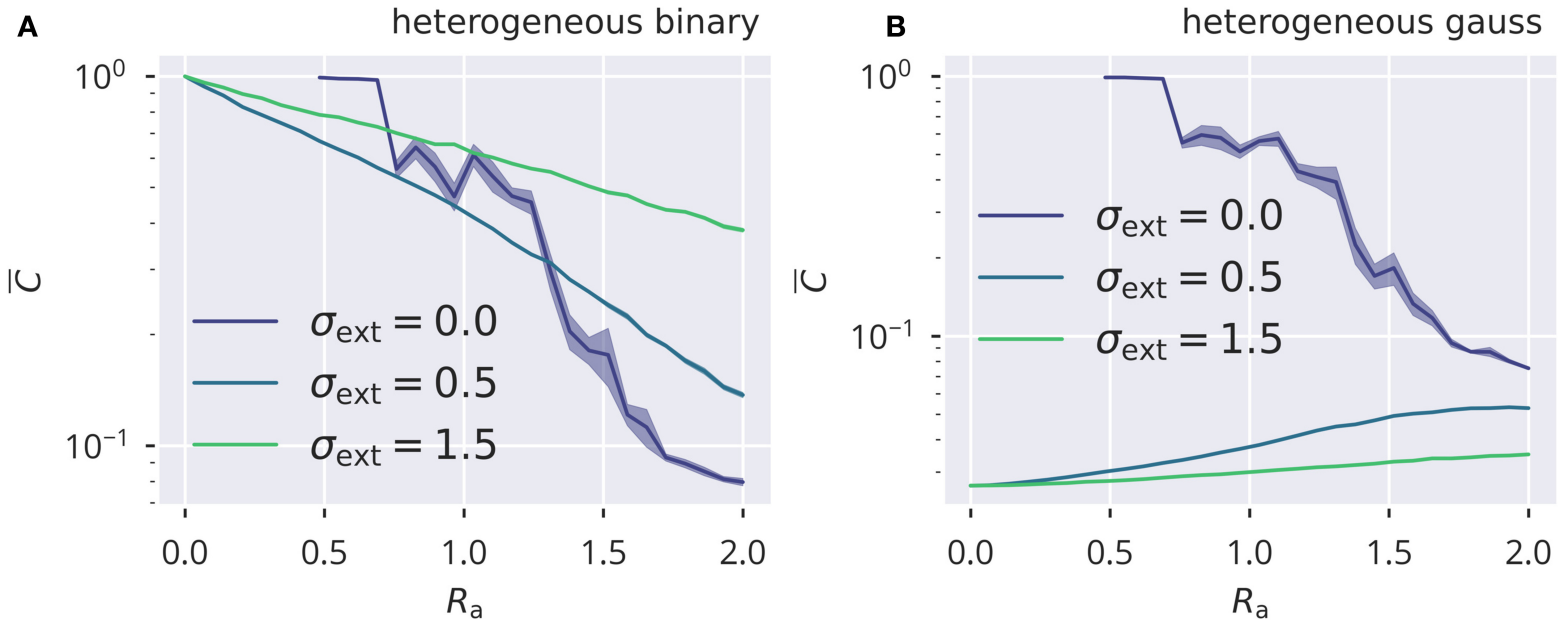
Input induced activity correlations. For heterogeneous binary and Gaussian inputs **(A,B)**, the dependency of mean activity cross correlations $\bar{C}$ (see Equation 21). $\bar{C}$ is shown as a function of the spectral radius *R*_a_. Results are obtained for *N* = 500 sites by averaging over five trials, with shadows indicating the standard error across trials. Correlations are due to finite-size effect for the autonomous case σ_ext_ = 0.

A crucial assumption leading to the proposed adaptation rules is the statistical independence of neural activity for describing the statistical properties of the bare recurrent contribution to the membrane potential, $x_{\text{bare}}=\underset{j}{\sum }W_{ij}y_{j}$. In particular, the variance $\sigma_{\text{bare}}^{2}$ of *x*_bare_ enters the mean-field approach described in section 5.6. Assuming statistical independence across the population for *y*_*i*_(*t*), it is simply given by $\sigma_{\text{bare}}^{2}=\sigma_{\text{w}}^{2}\sigma_{\text{y}}^{2}$, where

(20) $$\begin{array}{l} \sigma_{\text{w}}^{2}\equiv \text{Var}\left[\overset{N}{\underset{j=1}{\sum }}W_{ij}\right] \end{array}$$

is the variance of the sum of the bare afferent synaptic weights (see also section 5.1). Being a crucial element of the proposed rules, deviations from the prediction of $\sigma_{\text{bare}}^{2}$ would also negatively affect the precision of tuning the spectral radius. In Figure 5, a comparison of the deviations $\left|\sigma_{\text{bare}}^{2}-\sigma_{\text{w}}^{2}\sigma_{\text{y}}^{2}\right|$ is presented for the four input protocols introduced in section 5.3. For the Gaussian protocols, for which neurons receive statistically uncorrelated external signals, one observes that $\sigma_{\text{bare}}^{2}\to \sigma_{\text{w}}^{2}\sigma_{\text{y}}^{2}$ in the thermodynamic limit *N* → ∞ via a power law, which is to be expected when the presynaptic neural activities are decorrelated. On the other side, binary 0/1 inputs act synchronous on all sites, either with site-dependent or site-independent strengths (heterogeneous/homogeneous). Corresponding activity correlations are induced and a finite and only weakly size-dependent difference between $\sigma_{\text{bare}}^{2}$ and $\sigma_{\text{w}}^{2}\sigma_{\text{y}}^{2}$ shows up. Substantial corrections to the analytic theory are to be expected in this case. To this extend we measured the cross-correlation *C*(*y*_*i*_, *y*_*j*_), defined as

(21) $$\begin{array}{l} \;\bar{C}=\frac{1}{N(N-1)}\sum_{i\neq j}|C(y_{i},y_{j})|,\\ C(y_{i},y_{j})\;=\frac{\text{Cov}(y_{i},y_{j})}{\sqrt{\text{Cov}(y_{i},y_{i})\text{Cov}(y_{j},y_{j})}}, \end{array}$$

with the covariance given by Cov(*y*_*i*_, *y*_*j*_) = 〈(*y*_*i*_ − 〈*y*_*i*_〉_*t*_)(*y*_*j*_ − 〈*y*_*j*_〉_*t*_)〉_*t*_. For a system of *N* = 500 neurons the results for the averaged absolute correlation $\bar{C}$ are presented in Figure 6 (see Supplementary Figure 10 for homogeneous input protocols). Autonomous echo-state layers are in chaotic states when supporting a finite activity level, which implies that correlations vanish in the thermodynamic limit *N* → ∞. The case σ_ext_ = 0, as included in Figure 6, serves consequently as a yardstick for the magnitude of correlations that are due to the finite number of neurons.

Input correlations were substantially above the autonomous case for correlated binary inputs, with the magnitude of $\bar{C}$ decreasing when the relative contribution of the recurrent activity increased. This was the case for increasing *R*_a_. The effect was opposite for the Gaussian protocol, for which the input did not induce correlations, but contributed to decorrelating neural activity. In this case, the mean absolute correlation $\bar{C}$ was suppressed when the internal activity became small in the limit *R*_a_ → 0. For larger *R*_a_, the recurrent input gained more impact on neural activity relative to the external drive and thus drove $\bar{C}$ toward an amount of correlation that would be expected in the autonomous case.

## 3. Discussion

The mechanisms for tuning the spectral radius via a local homeostatic adaptation rule introduced in the present study require neurons to have the ability to distinguish and locally measure both external and recurrent input contributions. For flow control, neurons need to be able to compare the recurrent membrane potential with their own activity, as assumed in section 2.2. On the other hand, variance control directly measures the variance of the external input and derives the activity target variance accordingly. The limiting factor to a successful spectral radius control is the amount of cross-correlation induced by external driving statistics. As such, the functionality and validity of the proposed mechanisms depended on the ratio between external input, i.e., feed-forward or feedback connections, with respect to recurrent, or lateral connections. In general, it is not straightforward to directly connect experimental evidence regarding the ratio between recurrent and feed-forward contributions to the effects observed in the model. It is, however, worthwhile to note that the fraction of synapses associated with interlaminar loops and intralaminar lateral connections are estimated to make up roughly 50% (Binzegger et al., 2004). Relating this to our model, it implies that the significant interneural correlations that we observed when external input strengths were of the same order of magnitude as the recurrent inputs, can not generally be considered an artifact of biologically implausible parameter choices. Synchronization (Echeveste and Gros, 2016) is in fact a widely observed phenomenon in the brain (Usrey and Reid, 1999), with possible relevance for information processing (Salinas and Sejnowski, 2001).

On the other hand, correlations due to shared input reduces the amount of information that can be stored in the neural ensemble (Bell and Sejnowski, 1995). Maximal information is achieved if neural activities or spikes trains form an orthogonal ensemble (Földiak, 1990; Bell and Sejnowski, 1995; Tetzlaff et al., 2012). Furthermore, neural firing in cortical microcircuits was found to be decorrelated across neurons, even if common external input was present (Ecker et al., 2010), that is, under a common orientation tuning. Therefore, the correlation we observed in our network due to shared input might be significantly reduced by possible modifications/extensions of our model: First, a strict separation between inhibitory and excitatory nodes according to Dale's law might help actively decorrelating neural activity (Tetzlaff et al., 2012; Bernacchia and Wang, 2013). Second, if higher dimensional input was used, a combination of plasticity mechanisms in the recurrent and feed-forward connections could lead to the formation of an orthogonal representation of the input (Földiak, 1990; Bell and Sejnowski, 1995; Wick et al., 2010), leading to richer, “higher dimensional” activity patterns, i.e., a less dominant largest principal component. Ultimately, if these measures helped in reducing neural cross-correlations in the model, we thus would expect them to also increase the accuracy of the presented adaptation mechanisms. We leave these modifications to possible future research.

Overall, we found flow control to be generally more robust than variance control in the sense that, while still being affected by the amount of correlations within the neural reservoir, the task performance was less so prone to changes in the external input strength. Comparatively stable network performance could be observed, in spite of certain deviations from the desired spectral radius (see Figure 3). A possible explanation may be that flow control uses a distribution of samples from only a restricted part of phase space, that is, from the phase space regions that are actually visited or “used” for a given input. Therefore, while a spectral radius of unity ensures—statistically speaking—the desired scaling properties in all phase-space directions, it seem to be enough to control the correct scaling for the subspace of activities that is actually used for a given set of input patters. Variance control, on the other hand, relies more strictly on the assumptions that neural activities are statistical independent. In consequence, the desired results could only be achieved under a rather narrow set of input statistics (independent Gaussian input with small variance). In addition, the approximate expression derived for the nonlinear transformation appearing in the mean field approximation adds another layer of potential source of systematic error to the control mechanism. This aspect also speaks in favor of flow control, since its rules are mathematically more simple. In contrast to variance control, the stationarity condition stated in Equation (12) is independent of the actual nonlinear activation function used and could easily be adopted in a modified neuron model. It should be noted, however, that the actual target *R*_t_ giving optimal performance might then also be affected.

Interestingly, flow control distinguishes itself from a conventional local activity-target perspective of synaptic homeostasis: There is no predefined set point in Equation (5). This allows heterogeneities of variances of neural activity to develop across the network, while retaining the average neural activity at a fixed predefined level.

We would like to point out that, for all the results presented here, only stationary processes were used for generating the input sequences. Therefore, it might be worth considering the potential effects of non-stationary, yet bounded, inputs on the results in future work. It should be noted, however, that the temporal domain enters both adaptation mechanisms only in the form of trailing averages of first and second moments. As a consequence, we expect the issue of non-stationarity of external inputs to present itself simply as a trade-off between slower adaptation, i.e., longer averaging time scales, and the mitigation of the effects of non-stationarities. Slow adaptation is, however, completely in line with experimental results on the dynamics of synaptic scaling, which is taking place on the time scale of hours to days (Turrigiano et al., 1998; Turrigiano, 2008).

## 4. Conclusion

Apart from being relevant from a theoretical perspective, we propose that the separability of recurrent and external contributions to the membrane potential is an aspect that is potentially relevant for the understanding of local homeostasis in biological networks. While homeostasis in neural compartments has been a subject of experimental research (Chen et al., 2008), to our knowledge, it has not yet been further investigated on a theoretical basis, although it has been hypothesized that the functional segregation within the dendritic structure might also affect (among other intraneural dynamical processes) homeostasis (Narayanan and Johnston, 2012). The neural network model used in this study lacks certain features characterizing biological neural networks, like strict positivity of the neural firing rate or Dale's law, viz E-I balance (Trapp et al., 2018). Future research should therefore investigate whether the here presented framework of local flow control can be implemented within more realistic biological neural network models. A particular concern regarding our findings is that biological neurons are spiking. The concept of an underlying instantaneous firing rate is, strictly speaking, a theoretical construct, let alone the definition of higher moments, such as the “variance of neural activity.” It is however acknowledged that the variability of the neural activity is central for statistical inference (Echeveste et al., 2020). It is also important to note that real-world biological control mechanisms, e.g., of the activity, rely on physical quantities that serve as measurable correlates. A well-known example is the intracellular calcium concentration, which is essentially a linearly filtered version of the neural spike train (Turrigiano, 2008). On a theoretical level, Cannon and Miller showed that dual homeostasis can successfully control the mean and variance of this type of spike-averaging physical quantities (Cannon and Miller, 2017). An extension of the flow control to filtered spike trains of spiking neurons could be an interesting subject of further investigations. However, using spiking neuron models would have shifted the focus of our research toward the theory of liquid state machines (Maass et al., 2002; Maass and Markram, 2004), exceeding the scope of this publication. We therefore leave the extension to more realistic network/neuron models to future work.

## 5. Materials and Methods

### 5.1. Model

We implemented an echo state network with *N* neurons, receiving *D*_in_ inputs. The neural activity is *y*_*i*_ ∈ [−1, 1], *x*_*i*_ the membrane potential, *u*_*i*_ the input activities, *W*_*ij*_ the internal synaptic weights and *I*_*i*_ the external input received. The output layer will be specified later. The dynamics

(22) $$\begin{array}{l} x_{i}\left(t\right)=a_{i}\overset{N}{\underset{j=1}{\sum }}W_{ij}y_{j}\left(t-1\right)+I_{i}\left(t\right),\;y_{i}\left(t\right)\text{ =}\tanh\left(x_{i}\left(t\right)-b_{i}\right) \end{array}$$

is discrete in time, where the input *I*_*i*_ is treated instantaneously. A tanh-sigmoidal has been used as a nonlinear activation function.

The synaptic renormalization factor *a*_*i*_ in (22) can be thought of as a synaptic scaling parameter that neurons use to regulate the overall strength of the recurrent inputs. The strength of the inputs *I*_*i*_ is unaffected, which is biologically plausible if external and recurrent signals arrive at separate branches of the dendritic tree (Spruston, 2008).

The *W*_*ij*_ are the bare synaptic weights, with *a*_*i*_*W*_*ij*_ being the components of the effective weight matrix ${\hat{W}}_{\text{a}}$. Key to our approach is that the propagation of activity is determined by ${\hat{W}}_{\text{a}}$, which implies that the spectral radius of the effective, and not of the bare weight matrix needs to be regulated.

The bare synaptic matrix *W*_*ij*_ is sparse, with a connection probability *p*_r_ = 0.1. The non-zero elements are drawn from a Gaussian with standard deviation

(23) $$\begin{array}{l} \sigma =\frac{\sigma_{\text{w}}}{\sqrt{Np_{\text{r}}}}, \end{array}$$

and vanishing mean μ. Here *Np*_r_ corresponds to the mean number of afferent internal synapses, with the scaling $\sim 1/\sqrt{Np_{\text{r}}}$ enforcing size-consistent synaptic-weight variances. As discussed in the results section, we applied the following adaptation mechanisms:

(24) $$\begin{array}{l} b_{i}\left(t\right)=b_{i}\left(t-1\right)+\epsilon_{\text{b}}\left[y_{i}\left(t\right)-\mu_{\text{t}}\right] \end{array}$$

for the thresholds *b*_*i*_.

- adaptation of gains, using flow control:
  (25) $$\begin{array}{l} \;a_{i}(t)=a_{i}(t-1)[1+\epsilon_{\text{a}}\Delta R_{i}(t)],\\ \Delta R_{i}(t)=R_{\text{t}}^{2}|y_{i}(t-1){|}^{2}-|x_{\text{r},i}(t){|}^{2}. \end{array}$$
- adaptation of gains, with variance control:
  (26) $$\begin{array}{l} a_{i}\left(t\right)=a_{i}\left(t-1\right)+\epsilon_{\text{a}}\left[\sigma_{\text{t},i}^{2}\left(t\right)-{\left(y_{i}\left(t\right)-\mu_{i}^{\text{y}}\left(t\right)\right)}^{2}\right] \end{array}$$
  (27) $$\begin{array}{l} \sigma_{\text{t},i}^{2}\left(t\right)=1-\frac{1}{\sqrt{1+2R_{\text{t}}^{2}y_{i}{\left(t\right)}^{2}+2\sigma_{\text{ext},i}^{2}\left(t\right)}} \end{array}$$
  (28) $$\begin{array}{l} \mu_{i}^{\text{y}}\left(t\right)=\mu_{i}^{\text{y}}\left(t-1\right)+\epsilon_{\mu }\left[y_{i}\left(t\right)-\mu_{i}^{\text{y}}\left(t-1\right)\right] \end{array}$$
  (29) $$\begin{array}{l} \sigma_{\text{ext},i}^{2}\left(t\right)=\sigma_{\text{ext},i}^{2}\left(t-1\right)+\epsilon_{\sigma }\left[{\left(I_{i}\left(t\right)-\mu_{\text{ext},i}\left(t\right)\right)}^{2}-\sigma_{\text{ext},i}^{2}\left(t-1\right)\right] \end{array}$$
  (30) $$\begin{array}{l} \mu_{\text{ext},i}\left(t\right)=\mu_{\text{ext},i}\left(t-1\right)+\epsilon_{\mu }\left[I_{i}\left(t\right)-\mu_{\text{ext},i}\left(t-1\right)\right]. \end{array}$$

Note that Equations (28)–(30) have the same mathematical form

$$\begin{array}{l} \langle trail\rangle \left(t\right)=\langle trail\rangle \left(t-1\right)+\epsilon \left[\langle var\rangle \left(t\right)-\langle trail\rangle \left(t-1\right)\right] \end{array}$$

since they only serve as trailing averages that are used in the two main Equations (26) and (27).

For a summary of all model parameters (see Table 1).

**Table 1:** Standard values for model parameters.

**N**	***p*_r_**	**σ_w_**	**μ_t_**	**ϵ_b_**	**ϵ_a_**	**ϵ_μ_**	**ϵ_σ_**
500	0.1	1	0.05	10^−3^	10^−3^	10^−4^	10^−3^

### 5.2. Convergence Acceleration for Flow Control

**Figure 7:**
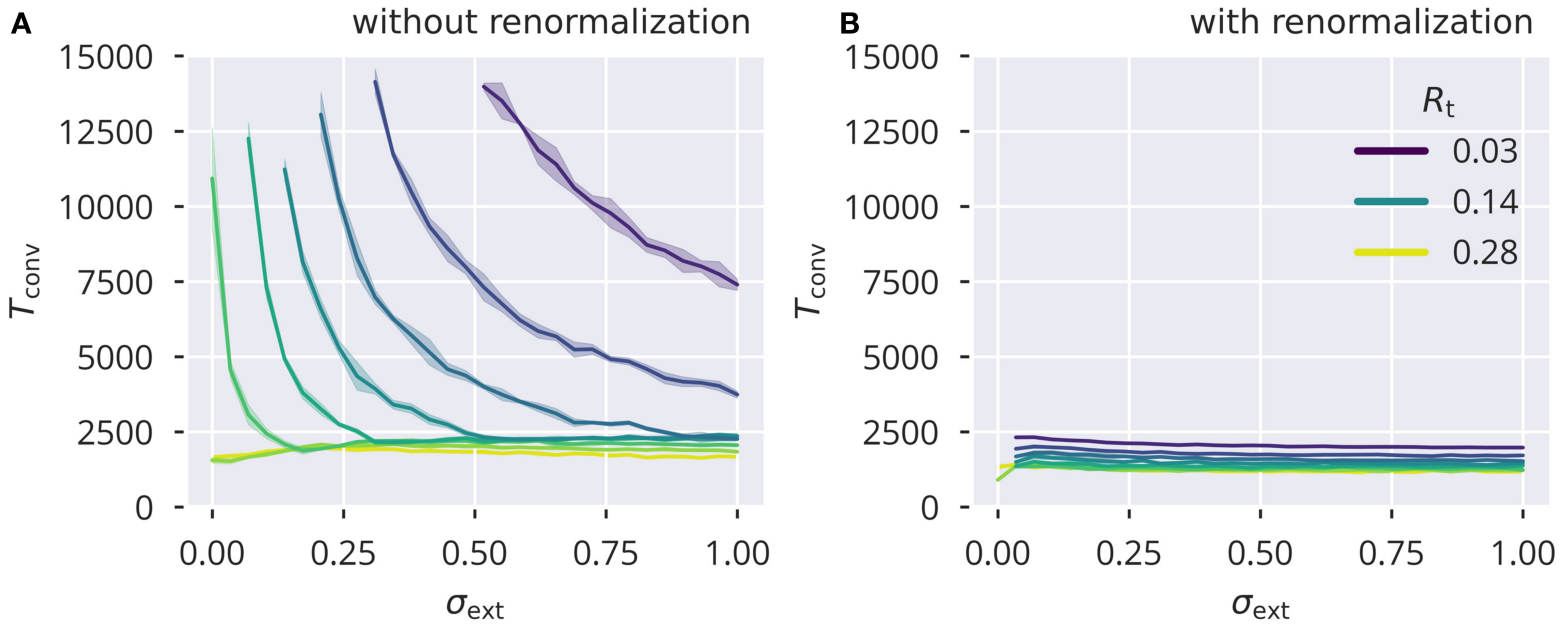
Convergence time with and without adaptation rate renormalization Number of time steps *T*_conv_ needed for $|R_{\text{a}}\left(t\right)-R_{\text{a}}\left(t-1\right){|}^{2}$ to fall below 10^−3^. Shown are results using heterogeneous Gaussian input without and with, **(A)** and respectively **(B)**, a renormalization of the learning rate $\epsilon_{a}\to \epsilon_{\text{a}}/{\bar{x}}_{\text{r}}^{2}$. Note that, due to computational complexity, an estimate of *R*_a_ given by (10) was used. An initial offset of 0.5 from the target *R*_t_ was used for all runs. Color coding of *R*_t_ is the same in both panels.

For small values of *R*_t_ and weak external input, the average square activities and membrane potentials $y_{i}^{2}\left(t\right)$ and $x_{\text{t},i}^{2}\left(t\right)$ can become very small. As a consequence, their difference entering Δ*R*_*i*_(*t*) in (25) also becomes small in absolute value, slowing down the convergence process. To eliminate this effect, we decided to rescale the learning rate by a trailing average of the squared recurrent membrane potential, i.e., $\epsilon_{a}\to \epsilon_{\text{a}}/{\bar{x}}_{\text{r}}^{2}$. The effect of this renormalization is shown in Figure 7. Rescaling the learning rate effectively removes the significant rise of convergence times for small σ_ext_ and small *R*_t_.

### 5.3. Input Protocols

Overall, we examined four distinct input protocols.

- Homogeneous Gaussian. Nodes receive inputs *I*_*i*_(*t*) that are drawn individually from a Gaussian with vanishing mean and standard deviation σ_ext_.
- Heterogeneous Gaussian. Nodes receive stochastically independent inputs *I*_*i*_(*t*) that are drawn from Gaussian distributions with vanishing mean and node specific standard deviations σ_*i*, ext_. The individual σ_*i*, ext_ are normal distributed, as drawn from the positive part of a Gaussian with mean zero and variance $\sigma_{\text{ext}}^{2}$.
- Homogeneous binary. Sites receive identical inputs *I*_*i*_(*t*) = σ_ext_*u*(*t*), where *u*(*t*) = ±1 is a binary input sequence.
- Heterogeneous binary. We define with
  (31) $$\begin{array}{l} I_{i}=W_{i}^{\text{u}}u\left(t\right),\;u_{j}\left(t\right)=\pm 1 \end{array}$$

the afferent synaptic weight vector $W_{i}^{\text{u}}$, which connects the binary input sequence *u*(*t*) to the network. All $W_{i}^{\text{u}}$ are drawn independently from a Gaussian with mean zero and standard deviation σ_ext_.

The Gaussian input variant simulates external noise. We used it in particular to test predictions of the theory developed in section 5.6. In order to test the performance of the echo state network with respect to the delayed XOR task, the binary input protocols are employed. A generalization of the here defined protocols to the case of higher-dimensional input signals would be straightforward.

### 5.4. Spectral Radius Adaptation Dynamics

**Figure 8:**
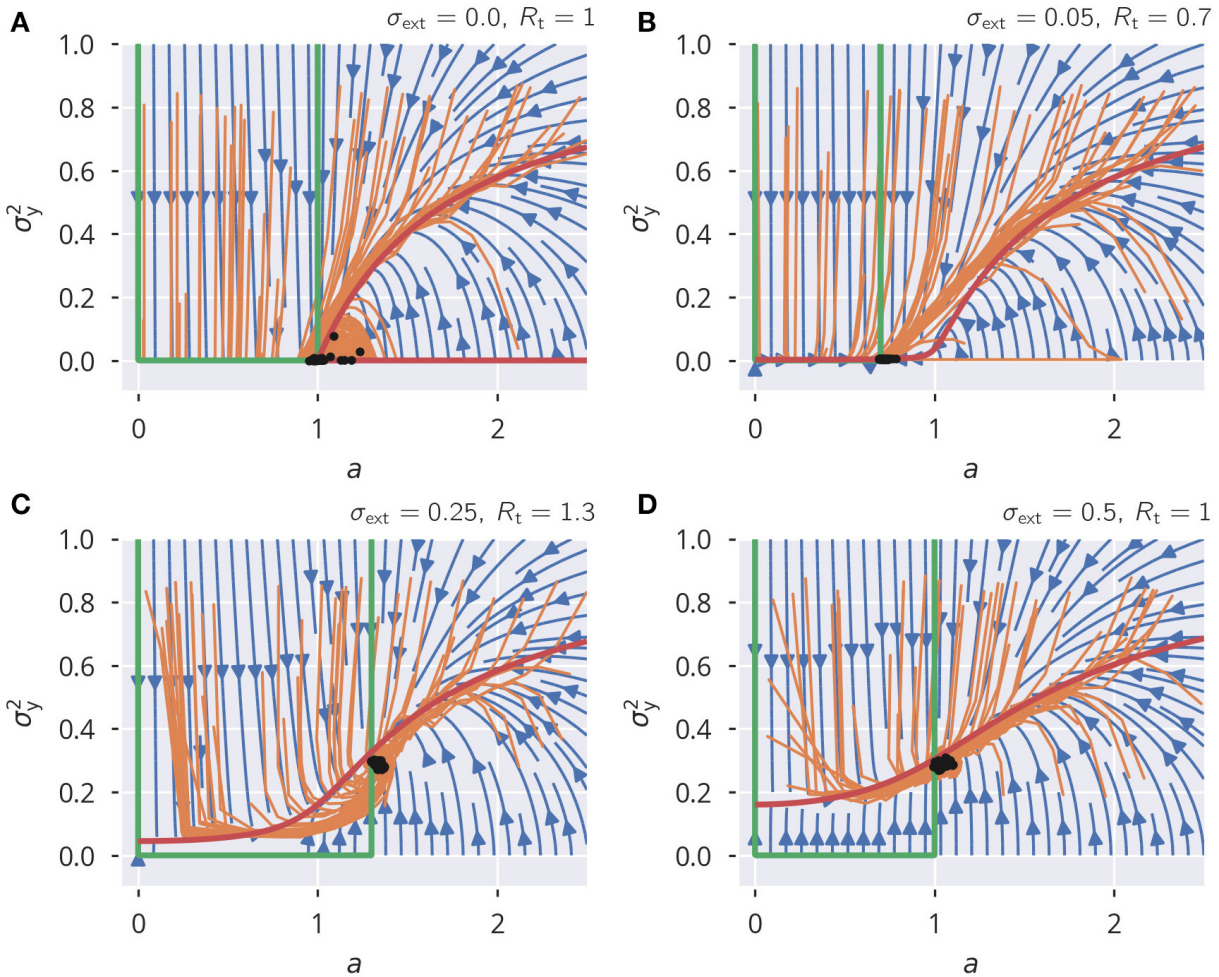
Spectral radius adaptation dynamics. The dynamics of the synaptic rescaling factor *a* and the squared activity $\sigma_{\text{y}}^{2}$ (orange), as given by (6). Also shown is the analytic approximation to the flow (blue), see (33) and (34), and the respective nullclines Δ*a* = 0 (green) and $\Delta \sigma_{\text{y}}^{2}=0$ (red). For the input, the heterogeneous binary protocol is used. **(A–D)** Correspond to different combinations of external input strengths and target spectral radii. The black dots show the stead-state configurations of the simulated systems. ϵ_a_ = 0.1.

For an understanding of the spectral radius adaptation dynamics of flow control, it is of interest to examine the effect of using the global adaptation constraint

(32) $$\begin{array}{l} \Delta R_{i}(t)=\frac{1}{N}[R_{t}^{2}||y(t-1){||}^{2}-||x_{r}(t){||}^{2}] \end{array}$$

in (5). The spectral radius condition (12) is then enforced directly, with the consequence that (32) is stable and precise even in the presence of correlated neural activities (see Supplementary Figures 5A–C). This rule, while not biologically plausible, provides an opportunity to examine the dynamical flow, besides the resulting state. There are two dynamic variables, *a* = *a*_*i*_ ∀ *i*, where, for the sake of simplicity, we assumed that all *a*_*i*_ are homogeneous, and the activity variance $\sigma_{\text{y}}^{2}=||\text{y}{||}^{2}/N$. The evolution of $\left(a,\sigma_{\text{y}}^{2}\right)$ resulting from the global rule (6) is shown in Figure 8. For the flow, Δ*a* = *a*(*t* + 1) − *a*(*t*) and $\Delta \sigma_{\text{y}}^{2}=\sigma_{\text{y}}^{2}\left(t\right)-\sigma_{\text{y}}^{2}\left(t-1\right)$, the approximation

(33) $$\begin{array}{l} \Delta a=\epsilon_{\text{a}}a\left(R_{\text{t}}^{2}-a^{2}\sigma_{\text{w}}^{2}\right)\sigma_{\text{y}}^{2} \end{array}$$

(34) $$\begin{array}{l} \Delta \sigma_{\text{y}}^{2}=1-\sigma_{\text{y}}^{2}-\frac{1}{\sqrt{1+2a^{2}\sigma_{\text{w}}^{2}\sigma_{\text{y}}^{2}+2\sigma_{\text{ext}}}} \end{array}$$

is obtained. For the scaling factor *a*, this leads to a fixed point of *R*_t_/σ_w_. We used the mean-field approximation for neural variances that is derived in section 5.6. The analytic flow compares well with numerics, as shown in Figure 8. For a subcritical rescaling factor *a* and σ_ext_ = 0, the system flows toward a line of fixpoints defined by a vanishing $\sigma_{\text{y}}^{2}$ and a finite *a* ∈ [0, 1] (see Figure 8A). When starting with *a* > 0, the fixpoint is instead $\left(a,\sigma_{\text{y}}^{2}\right)=\left(1,0\right)$. The situation changes qualitatively for finite external inputs, viz when σ_ext_ > 0, as shown in Figures 8B–D. The nullcline $\Delta \sigma_{\text{y}}^{2}=0$ is now continuous and the system flows to the fixed point, as shown in Figures 8B–D, with the value of $\sigma_{\text{y}}^{2}$ being determined by the intersection of the two nullclines. In addition, we also varied the target spectral radius (see Figures 8B,C). This caused a slight mismatch between the flow of the simulated systems and the analytic flow. It should be noted, however, that this is to be expected anyhow since we used an approximation for the neural variances, again (see section 5.6).

This analysis shows that external input is necessary for a robust flow toward the desired spectral weight, the reason being that the dynamics dies out before the spectral weight can be adapted when the isolated systems starts in the subcritical regime.

### 5.5. Extended Theory of Flow Control for Independent Neural Activity

We would like to show that the stationarity condition in Equation (12) results in the correct spectral radius, under the special case of independently identically distributed neural activities with zero mean.

We start with Equation (12) as a stationarity condition for a given *R*_t_:

(35) $$\begin{array}{l} {\text{〈 ||}x_{\text{r}}(t){||}^{2}\rangle }_{\text{t}}\overset{!}{=}R_{\text{t}}^{2}{\text{〈||}y(t-1){||}^{2}\rangle }_{\text{t}}\text{​}. \end{array}$$

We can express the left side of the equation as

(36) $$\begin{array}{l} E{\left[{\text{y}}^{†}\left(t\right){\hat{W}}_{\text{a}}^{†}{\hat{W}}_{\text{a}}\text{y}\left(t\right)\right]}_{t}. \end{array}$$

We define ${\hat{U}}_{\text{a}}\text{ ≡=}{\hat{W}}_{\text{a}}^{†}{\hat{W}}_{\text{a}}$ with $\left\{\sigma_{k}^{2}\right\}$ being the set of eigenvalues, which are also the squared singular values of ${\hat{W}}_{\text{a}}$, and {**u**_*k*_} the respective set of orthonormal (column) eigenvectors. We insert the identity $\overset{N}{\underset{k=1}{\sum }}{\text{u}}_{k}{\text{u}}_{k}^{†}$ and find

(37) $$\begin{array}{l} E{\left[{\text{y}}^{†}\left(t\right){\hat{U}}_{\text{a}}\overset{N}{\underset{k=1}{\sum }}{\text{u}}_{k}{\text{u}}_{k}^{†}\text{y}\left(t\right)\right]}_{t} \end{array}$$

(38) $$\begin{array}{l} =\text{E}{\left[\sum_{k=1}^{N}\sigma_{k}^{2}y^{†}(t)u_{k}u_{k}^{†}y(t)\right]}_{t} \end{array}$$

(39) $$\begin{array}{l} =\sum_{k=1}^{N}\sigma_{k}^{2}u_{k}^{†}\text{E}{\left[y(t)y^{†}(t)\right]}_{t}u_{k} \end{array}$$

(40) $$\begin{array}{l} =\sum_{k=1}^{N}\sigma_{k}^{2}u_{k}^{†}{\hat{C}}_{\text{yy}}u_{k} \end{array}$$

(41) $$\begin{array}{l} =\text{Tr}\left({\hat{D}}_{\sigma^{2}}{\hat{S}}_{\text{u}}^{†}{\hat{C}}_{\text{yy}}{\hat{S}}_{\text{u}}\right)\text{​}. \end{array}$$

Given zero mean neural activity, ${\hat{C}}_{\text{yy}}=\text{E}{\left[\text{y}\left(t\right){\text{y}}^{†}\left(t\right)\right]}_{t}$ is the covariance matrix of neural activities. ${\hat{D}}_{\sigma^{2}}$ is a diagonal matrix holding the $\left\{\sigma_{k}^{2}\right\}$ and ${\hat{S}}_{\text{u}}$ is a unitary matrix whose columns are {**u**_*k*_}. ${\hat{S}}_{\text{u}}^{†}{\hat{C}}_{\text{yy}}{\hat{S}}_{\text{u}}$ is expressing ${\hat{C}}_{\text{yy}}$ in the diagonal basis of ${\hat{U}}_{\text{a}}$.

Including the right hand side of (35), we get

(42) $$\begin{array}{l} \text{Tr}\left({\hat{D}}_{\sigma^{2}}{\hat{S}}_{\text{u}}^{†}{\hat{C}}_{\text{yy}}{\hat{S}}_{\text{u}}\right)=R_{\text{t}}^{2}\text{Tr}\left({\hat{C}}_{\text{yy}}\right). \end{array}$$

However, since the trace is invariant under a change of basis, we find

(43) $$\begin{array}{l} \text{Tr}\left({\hat{D}}_{\sigma^{2}}{\hat{S}}_{\text{u}}^{†}{\hat{C}}_{\text{yy}}{\hat{S}}_{\text{u}}\right)=R_{\text{t}}^{2}\text{Tr}\left({\hat{S}}_{\text{u}}^{†}{\hat{C}}_{\text{yy}}{\hat{S}}_{\text{u}}\right). \end{array}$$

Defining ${\hat{C}}^{\text{u}}\text{ ≡ =}{\hat{S}}_{\text{u}}^{†}{\hat{C}}_{\text{yy}}{\hat{S}}_{\text{u}}$, we get

(44) $$\begin{array}{l} \overset{N}{\underset{k=1}{\sum }}\sigma_{k}^{2}C_{kk}^{\text{u}}=R_{\text{t}}^{2}\overset{N}{\underset{k=1}{\sum }}C_{kk}^{\text{u}}. \end{array}$$

If we assume that the node activities are independently identically distributed with zero mean, we get ${\left({\hat{C}}_{\text{yy}}\right)}_{ij}={\left({\hat{C}}^{\text{u}}\right)}_{ij}={\langle y^{2}\rangle }_{t}\delta_{ij}$. In this case, which was also laid out in section 2.6, the equation reduces to

(45) $$\begin{array}{l} \overset{N}{\underset{k=1}{\sum }}\sigma_{k}^{2}=R_{\text{t}}^{2}N. \end{array}$$

The Frobenius norm of a square Matrix $\hat{A}$ is given by ${∥\hat{A}∥}_{\text{F}}^{2}\equiv \underset{i,j}{\sum }{\hat{A}}_{ij}^{2}$. Furthermore, the Frobenius norm is linked to the singular values via ${∥\hat{A}∥}_{\text{F}}^{2}=\underset{k}{\sum }\sigma_{k}^{2}\left(\hat{A}\right)$ (Sengupta and Mitra, 1999; Shen, 2001). This allows us to state

(46) $$\begin{array}{l} \underset{i,j}{\sum }{\left({\hat{W}}_{\text{a}}\right)}_{ij}^{2}=R_{\text{t}}^{2}N \end{array}$$

which, by using (10), gives

(47) $$\begin{array}{l} R_{\text{a}}^{2}=R_{\text{t}}^{2}. \end{array}$$

A slightly less restrictive case is that of uncorrelated but inhomogeneous activity, that is ${\left({\hat{C}}_{\text{yy}}\right)}_{ij}={\langle y_{i}^{2}\rangle }_{t}\delta_{ij}$. The statistical properties of the diagonal elements $C_{kk}^{\text{u}}$ then determine to which degree one can still claim that Equation (44) leads to Equation (45). Supplementary Figure 11 shows an example of a randomly generated realization of ${\left({\hat{C}}_{\text{yy}}\right)}_{ij}={\langle y_{i}^{2}\rangle }_{t}$ and the resulting diagonal elements of ${\overset{\text{\textasciicircum{}}}{C}}^{\text{u}}$, where the corresponding orthonormal basis ${\hat{S}}_{\text{u}}$ was generated from the SVD of a random Gaussian matrix. As one can see, the basis transformation has a strong smoothing effect on the diagonal entries, while the mean over the diagonal elements is preserved. Note that this effect was not disturbed by introducing random row-wise multiplications to the random matrix from which the orthonormal basis was derived. The smoothing of the diagonal entries allows us to state that $C_{kk}^{\text{u}}≊\langle y^{2}\rangle$ is a very good approximation in the case considered, which therefore reduces (44) to the homogeneous case previously described. We can conclude that the adaptation mechanism also gives the desired spectral radius under uncorrelated inhomogeneous activity.

In the most general case, we can still state that if $C_{kk}^{\text{u}}$ and $\sigma_{k}^{2}$ are uncorrelated, for large *N*, Equation (44) will tend toward

(48) $$\begin{array}{l} N\langle \sigma^{2}\rangle \langle C^{\text{u}}\rangle =NR_{\text{t}}^{2}\langle C^{\text{u}}\rangle \end{array}$$

which would also lead to Equation (45). However, we can not generally guarantee statistical independence since the recurrent contribution on neural activities and the resulting entries of ${\hat{C}}_{\text{yy}}$ and thus also $C_{kk}^{\text{u}}$ are linked to $\hat{S}$ and $\sigma_{k}^{2}$, being the SVD of the recurrent weight matrix.

### 5.6. Mean Field Theory for Echo State Layers

In the following, we deduce analytic expressions allowing to examine the state of echo-state layers subject to a continuous timeline of inputs. Our approach is similar to the one presented by Massar and Massar (2013).

The recurrent part of the input *x*_*i*_ received by a neuron is a superposition of *Np*_r_ terms, which are assumed here to be uncorrelated. Given this assumption, the self-consistency equations

(49) $$\begin{array}{l} \sigma_{\text{y},i}^{2}=\int_{-\infty }^{\infty }\mathrm{dx}\tanh^{2}(x)N_{\mu_{i},\sigma_{i}}(x)-\mu_{\text{y},i}^{2} \end{array}$$

(50) $$\begin{array}{l} \mu_{\text{y},i}=\int_{-\infty }^{\infty }\text{dx }\tanh(x)N_{\mu_{i},\sigma_{i}}(x) \end{array}$$

(51) $$\begin{array}{l} \sigma_{i}^{2}=a_{i}^{2}\sigma_{\text{w}}^{2}{\langle \sigma_{\text{y},j}^{2}\rangle }_{j}+\sigma_{\text{ext},i}^{2},\;\mu_{i}=\mu_{\text{ext},i}-b_{i} \end{array}$$

determine the properties of the stationary state. We recall that σ_w_ parameterizes the distribution of bare synaptic weights via (23). The general expressions (49) and (50) hold for all neurons, with the site-dependency entering exclusively via *a*_*i*_, *b*_*i*_, σ_ext, *i*_ and μ_ext, *i*_, as in (51), with the latter characterizing the standard deviation and the mean of the input. Here, $a_{i}^{2}\sigma_{\text{w}}^{2}\sigma_{\text{y}}^{2}$ is the variance of the recurrent contribution to the membrane potential, *x*, and σ^2^ the respective total variance. The membrane potential is Gaussian distributed, as *N*_μ, σ_(*x*), with mean μ and variance σ^2^, which are both to be determined self-consistently. Variances are additive for stochastically independent processes, which has been assumed in (51) to be the case for recurrent activities and the external inputs. The average value for the mean neural activity is μ_*i*_.

For a given set of *a*_*i*_, σ_ext, *i*_, and *b*_*i*_, the means and variances of neural activities, $\sigma_{\text{y},i}^{2}$ and μ_y, *i*_, follow implicitly.

**Figure 9:**
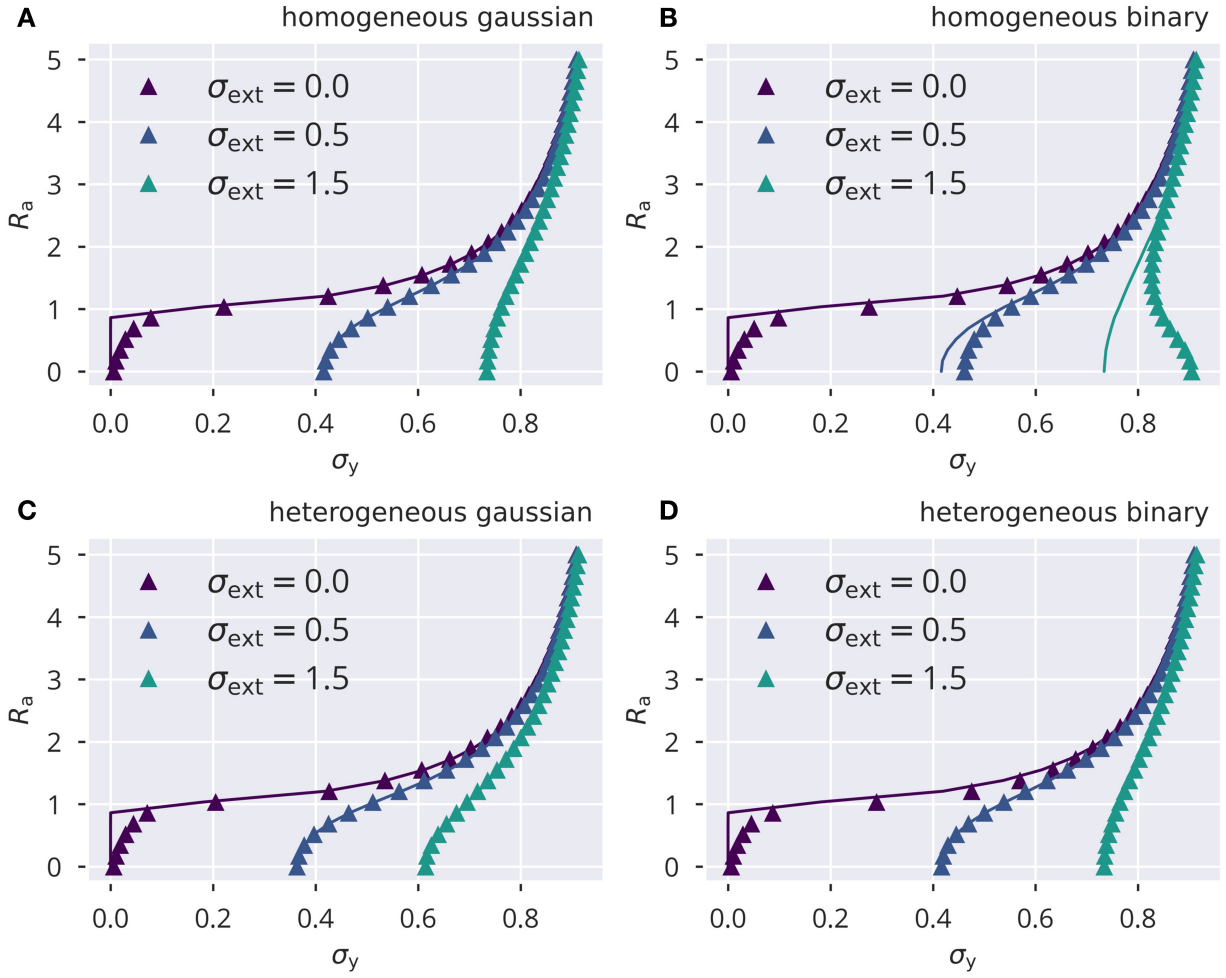
Variance control for the spectral radius. The spectral radius *R*_a_, given by the approximation $R_{\text{a}}^{2}=\underset{i}{\sum }a_{i}^{2}/N$, for the four input protocols defined in section 5.3. Lines show the numerical self-consistency solution of (49), symbols the full network simulations. Note the instability for small σ_y_ and σ_ext_. **(A)** Homogeneous independent Gaussian input. **(B)** Homogeneous identical binary input. **(C)** Heterogeneous independent Gaussian input. **(D)** Heterogeneous identical binary input.

We compared the numerically determined solutions of (49) and (50) against full network simulations using, as throughout this study, *N* = 500, *p*_r_ = 0.1, σ_w_ = 1, μ_t_ = 0.05. In Figure 9, the spectral radius *R*_a_ is given for the four input protocols defined in section 5.3. The identical ensemble of input standard deviations σ_ext, *i*_ enters both theory and simulations. Theory and simulations are in good accordance for vanishing input. Here, the reason is that finite activity levels are sustained in an autonomous random neural network when the ongoing dynamics is chaotic and hence decorrelated. For reduced activity levels, viz for small variances $\sigma_{\text{y}}^{2}$, the convergence of the network dynamics is comparatively slow, which leads to a certain discrepancy with the analytic prediction (see Figure 9).

#### 5.6.1. Gaussian Approximation

The integral occurring in the self-consistency condition (49) can be evaluated explicitly when a tractable approximation to the squared transfer function tanh^2^() is available. A polynomial approximation would capture the leading behavior close to the origin, however without accounting for the fact that tanh^2^() converges to unity for large absolute values of the membrane potential. Alternatively, an approximation incorporating both conditions, the correct second-order scaling for small, and the correct convergence for large arguments, is given by the Gaussian approximation

(52) $$\begin{array}{l} \tanh^{2}\left(x\right)\approx 1-\exp\left(-x^{2}\right). \end{array}$$

With this approximation the integral in (49) can be evaluated explicitly. The result is

(53) $$\begin{array}{l} \frac{1}{1-\sigma_{\text{y}}^{2}-\mu_{\text{y}}^{2}}=\sqrt{1+2\sigma^{2}}/\exp\left(-\mu^{2}/\left(1+2\sigma^{2}\right)\right)\\ \text{​​​​​​​ ​​}=\sqrt{1+2a^{2}\sigma_{\text{w}}^{2}\sigma_{\text{y}}^{2}+2\sigma_{\text{ext}}^{2}}/\\ \;\exp\left(-\mu^{2}/\left(1+2a^{2}\sigma_{\text{w}}^{2}\sigma_{\text{y}}^{2}+2\sigma_{\text{ext}}^{2}\right)\right). \end{array}$$

Assuming that μ ≈ 0 and μ_y_ ≈ 0, inverting the first equation yields a relatively simple analytic approximation for the variance self-consistency equation:

(54) $$\begin{array}{l} \sigma_{\text{y}}^{2}=1-\frac{1}{\sqrt{1+2a^{2}\sigma_{\text{w}}^{2}\sigma_{\text{y}}^{2}+2\sigma_{\text{ext}}^{2}}}. \end{array}$$

This equation was then used for the approximate update rule in (8) and (34).

Alternatively, we can write (54) as a self- consistency equation between $\sigma_{\text{y}}^{2}$, $\sigma_{\text{ext}}^{2}$ $a^{2}\sigma_{\text{w}}^{2}=R_{\text{a}}^{2}$, describing a phase transition at *R*_a_ = 1:

(55) $$\begin{array}{l} 2R_{\text{a}}^{2}\sigma_{\text{y}}^{2}{\left(1-\sigma_{\text{y}}^{2}\right)}^{2}=1-\left(1+2\sigma_{\text{ext}}^{2}\right){\left(1-\sigma_{\text{y}}^{2}\right)}^{2}. \end{array}$$

**Figure 10:**
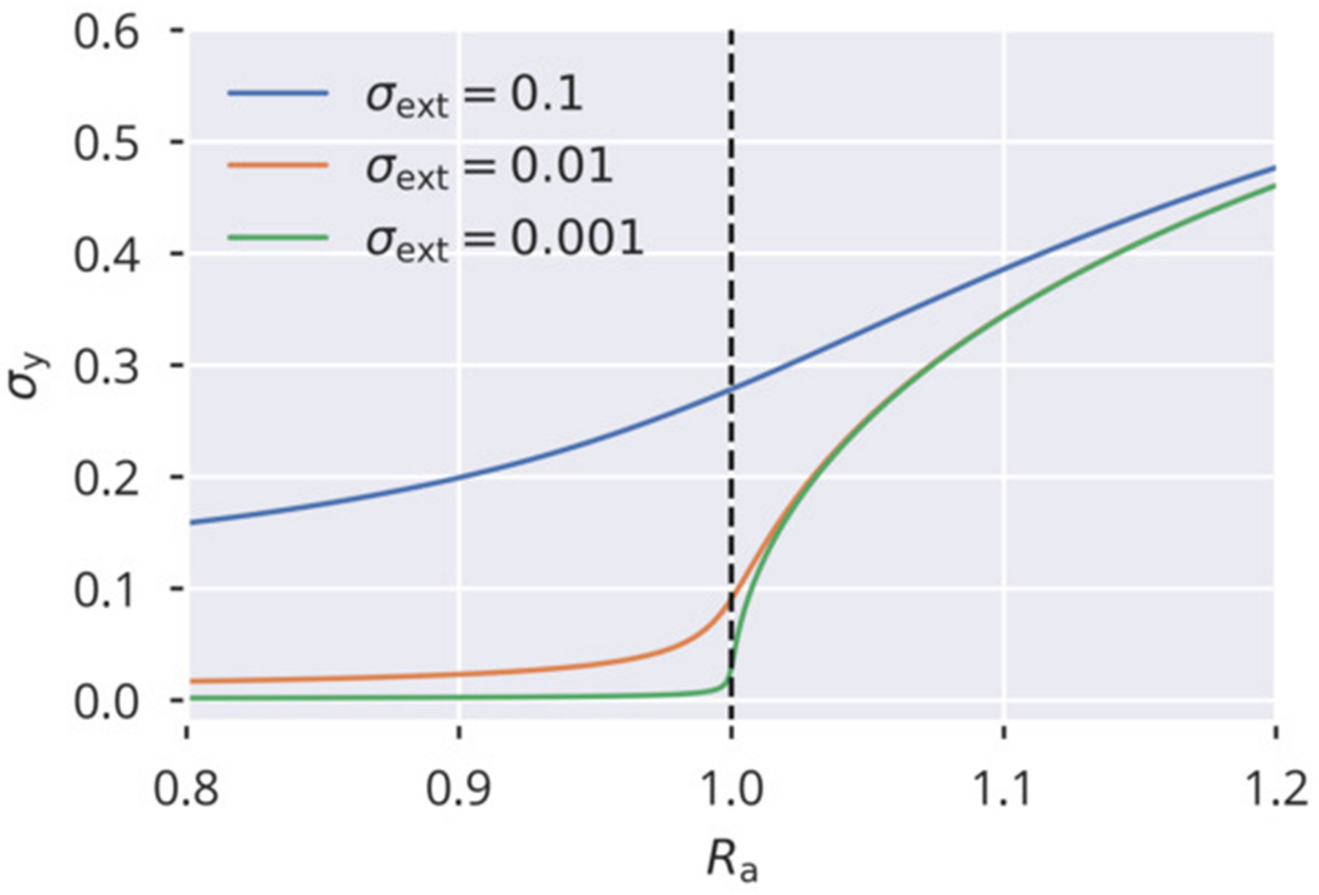
Phase transition of activity variance Shown are solutions of the analytical approximation given in (55), capturing the onset of activity (characterized by its variance $\sigma_{\text{y}}^{2}$) at the critical point *R*_a_ = 1.

See Figure 10 for solutions of (55) for different values of $\sigma_{\text{ext}}^{2}$. Note that for vanishing external driving and values of *R*_a_ above but close to the critical point, the standard deviation σ_y_ scales with $\sigma_{\text{y}}\propto {\left(R_{\text{a}}-1\right)}^{1/2}$, which is the typical critical exponent for the order parameter in classical Landau theory of second-order phase transitions (Gros, 2008, p. 169). If combined with a slow homeostatic process, flow or variance control in our case, this constitutes a system with an absorbing phase transition (Gros, 2008, p. 182–183), settling at the critical point *R*_a_ = 1. This phase transition can also be observed in Figure 9 for σ_ext_ = 0 as a sharp onset in σ_y_.

## Data Availability Statement

Publicly available datasets were analyzed in this study. This data can be found here: The datasets generated for this study can be found at: https://itp.uni-frankfurt.de/~fschubert/data_esn_frontiers. Simulation and plotting code is available at: https://github.com/FabianSchubert/ESN_Frontiers.

## Author Contributions

FS and CG contributed equally to the writing and review of the manuscript. FS provided the code, ran the simulations, and prepared the figures. All authors contributed to the article and approved the submitted version.

## Conflict of Interest

The authors declare that the research was conducted in the absence of any commercial or financial relationships that could be construed as a potential conflict of interest.
