# Supplementary material for: Local Homeostatic Regulation of the Spectral Radius of Echo-State Networks
Source: Front Comput Neurosci. 2021 Feb 24;15:587721. doi: 10.3389/fncom.2021.587721 (PMC7958921; doi:10.3389/fncom.2021.587721)
Supplement: Supplementary file 1 [file Data_Sheet_1.PDF]

Supplementary Material

1 SUPPLEMENTARY FIGURES

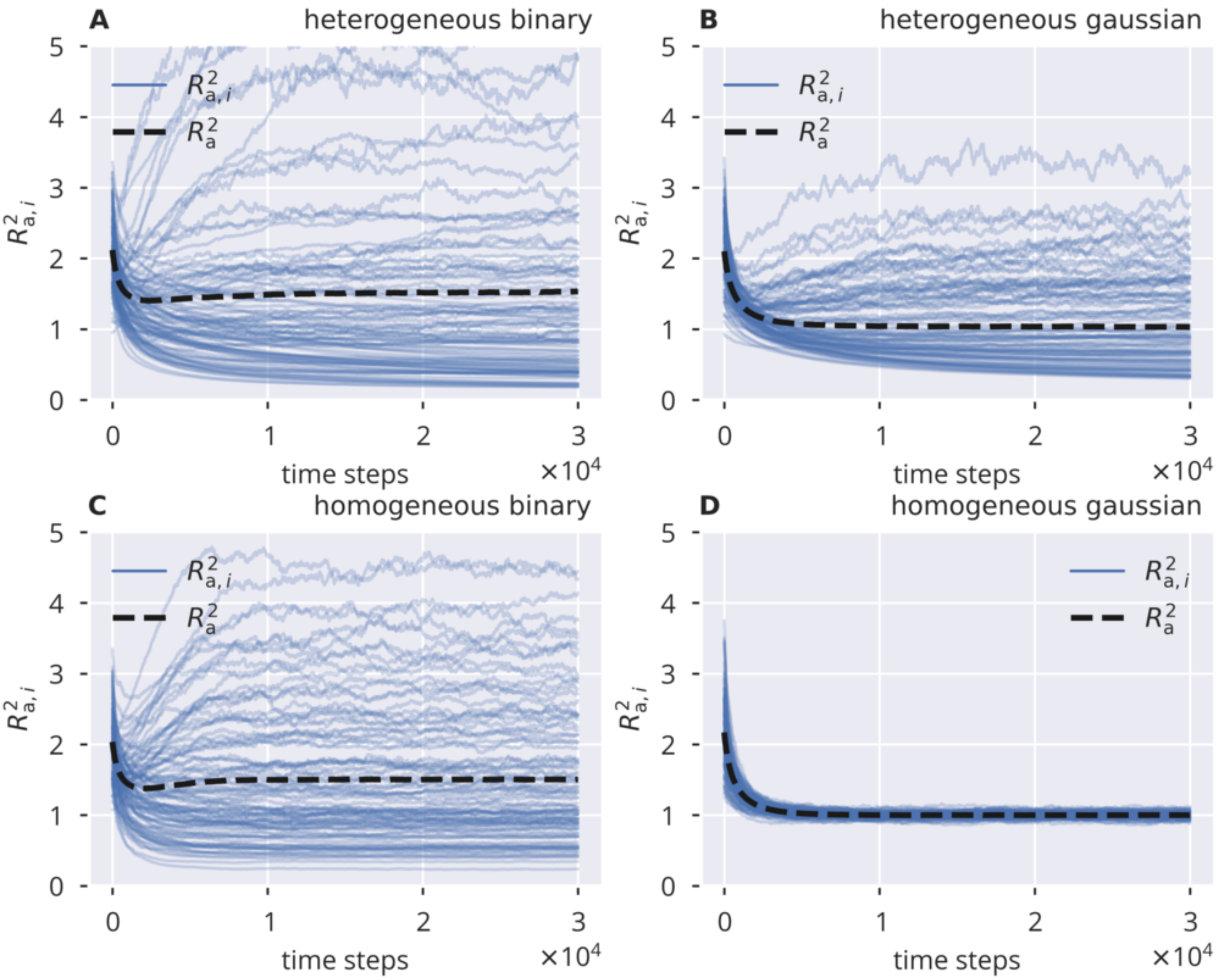

Figure S1. Adaptation dynamics, flow control, local Panels **A–D** show the dynamics of the square spectral radius R_a^2 and local estimates $R_{a,i}^2$ under local flow control for different input protocols, as given in the panel titles.

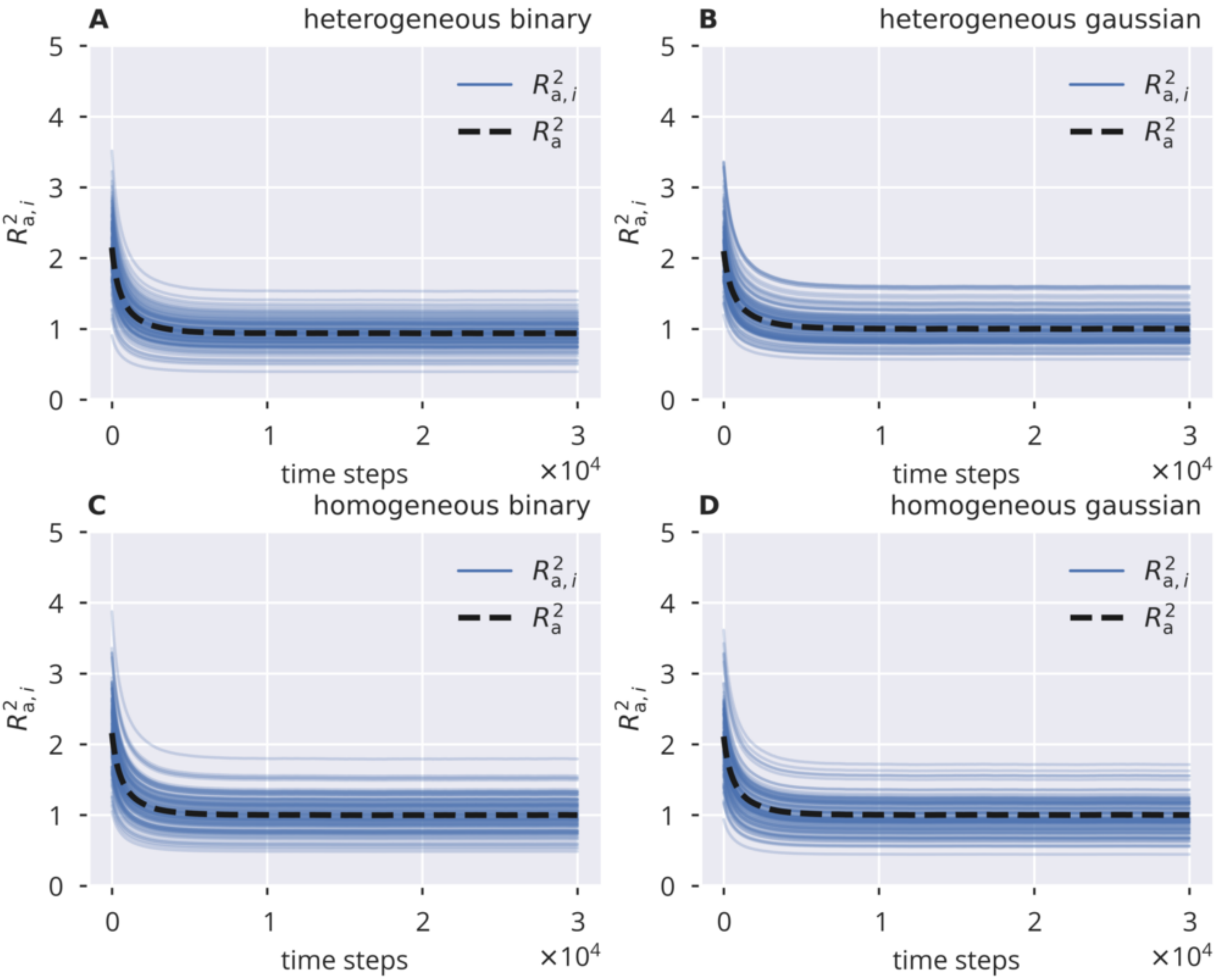

Figure S2. Adaptation dynamics, flow control, global Panels **A–D** show the dynamics of the square spectral radius R^2_{a} and local estimates $R^2_{\mathrm{a},i}$ under global flow control for different input protocols, as given in the panel titles.

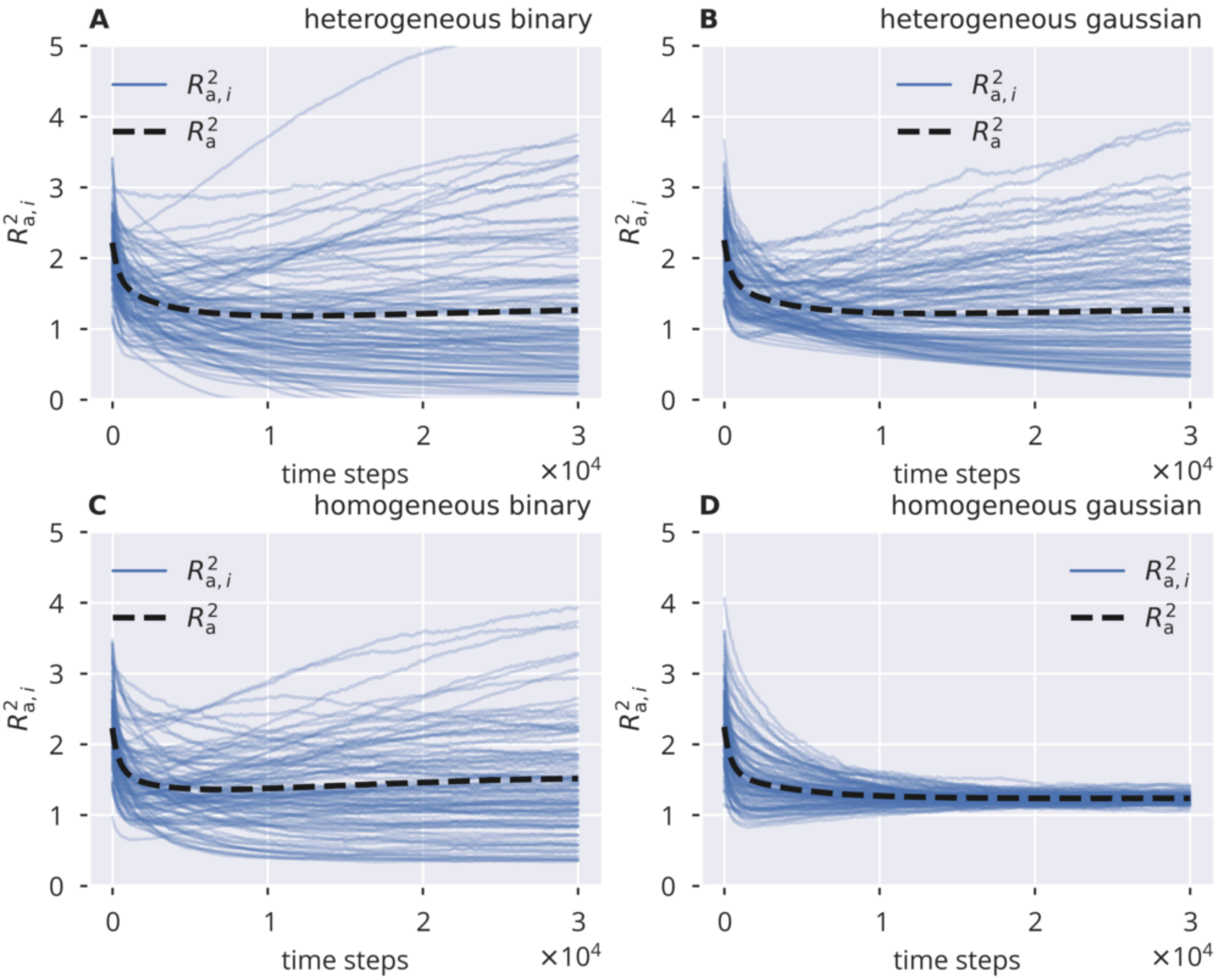

Figure S3. Adaptation dynamics, variance control, local Panels **A–D** show the dynamics of the square spectral radius R_a^2 and local estimates $R_{a,i}^2$ under local variance control for different input protocols, as given in the panel titles.

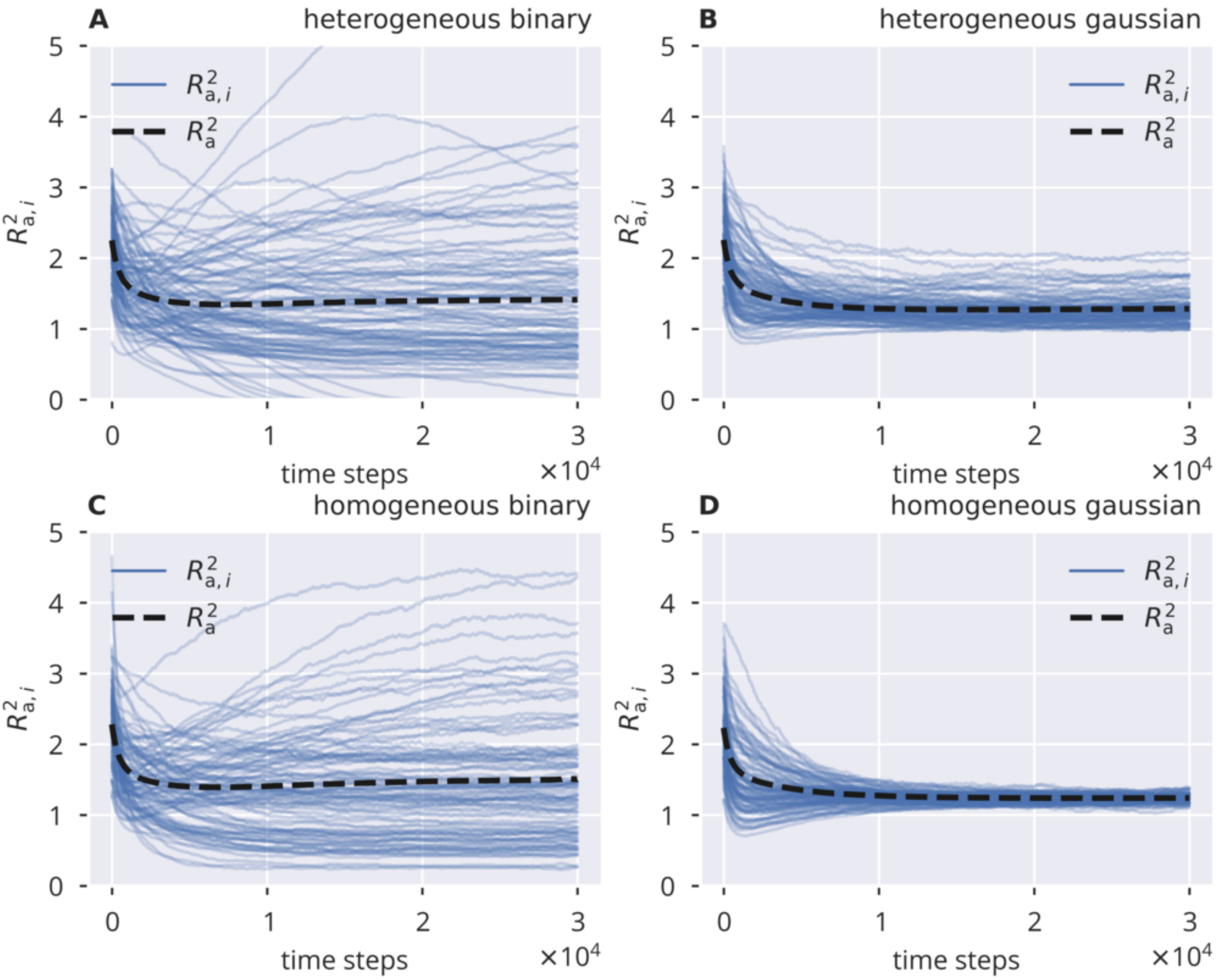

Figure S4. Adaptation dynamics, variance control, global Panels **A–D** show the dynamics of the square spectral radius R_a^2 and local estimates $R_{a,i}^2$ under global variance control for different input protocols, as given in the panel titles.

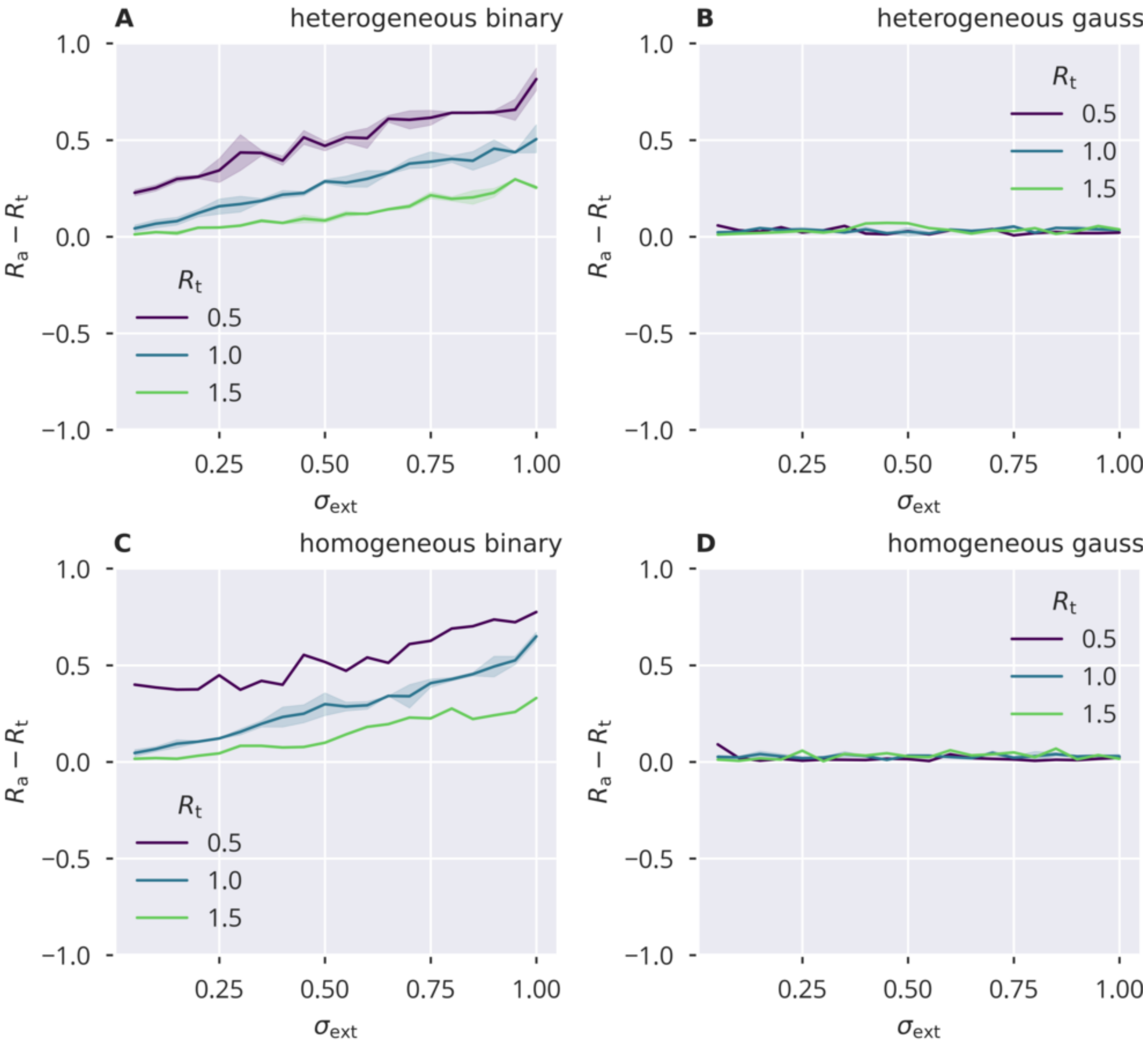

Figure S5. Difference of the spectral radius after adaptation and the target spectral radius, flow control For different standard deviations σ_{ext} of the external input, the error $R_\text{a} - R_\text{t}$ between the resulting spectral radius R_a and the target spectral radius $R_\text{t}$was determined. Heterogeneous/homogeneous binary input (**A**/**C**) led to positive deviations from the target spectral radius for stronger external input. Heterogeneous/homogeneous Gaussian input (**B**/**D**) yielded perfect alignment between R_a and R_t. Local adaptation was used for both panels.

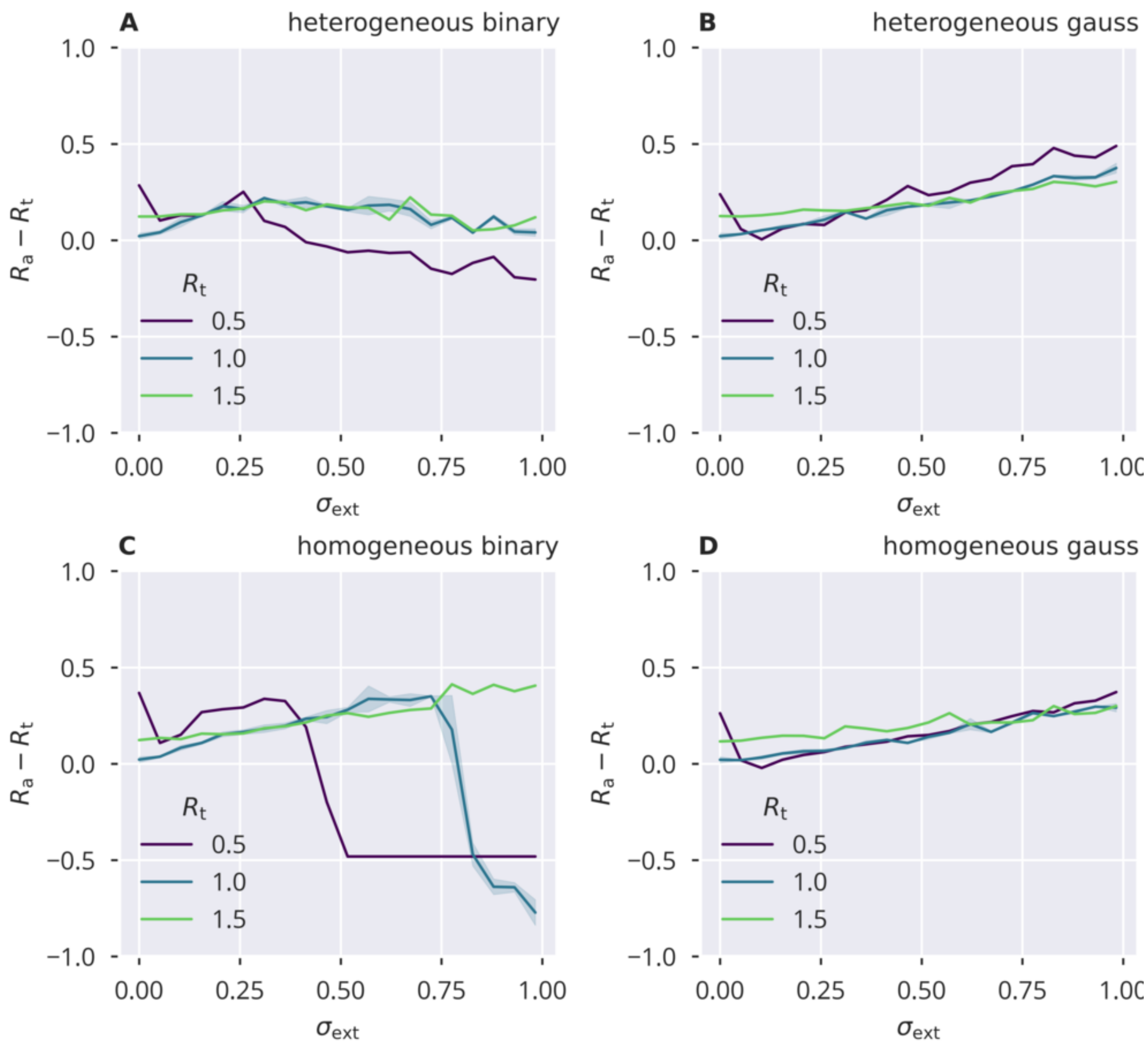

Figure S6. Difference of the spectral radius after adaptation and the target spectral radius, variance control For different standard deviations σ_{ext} of the external input, the error $R_{\text{a}} - R_{\text{t}}$ between the resulting spectral radius R_{a} and the target spectral radius $R_{\text{t}}$was determined. Heterogeneous binary input (**A**) led to a good alignment. On the other hand, homogeneous binary input (**C**) caused strong deviation from the target for larger input. Heterogeneous/homogeneous Gaussian input (**B/D**) both resulted in positive deviations that increased for larger input strengths. Local adaptation was used for both panels.

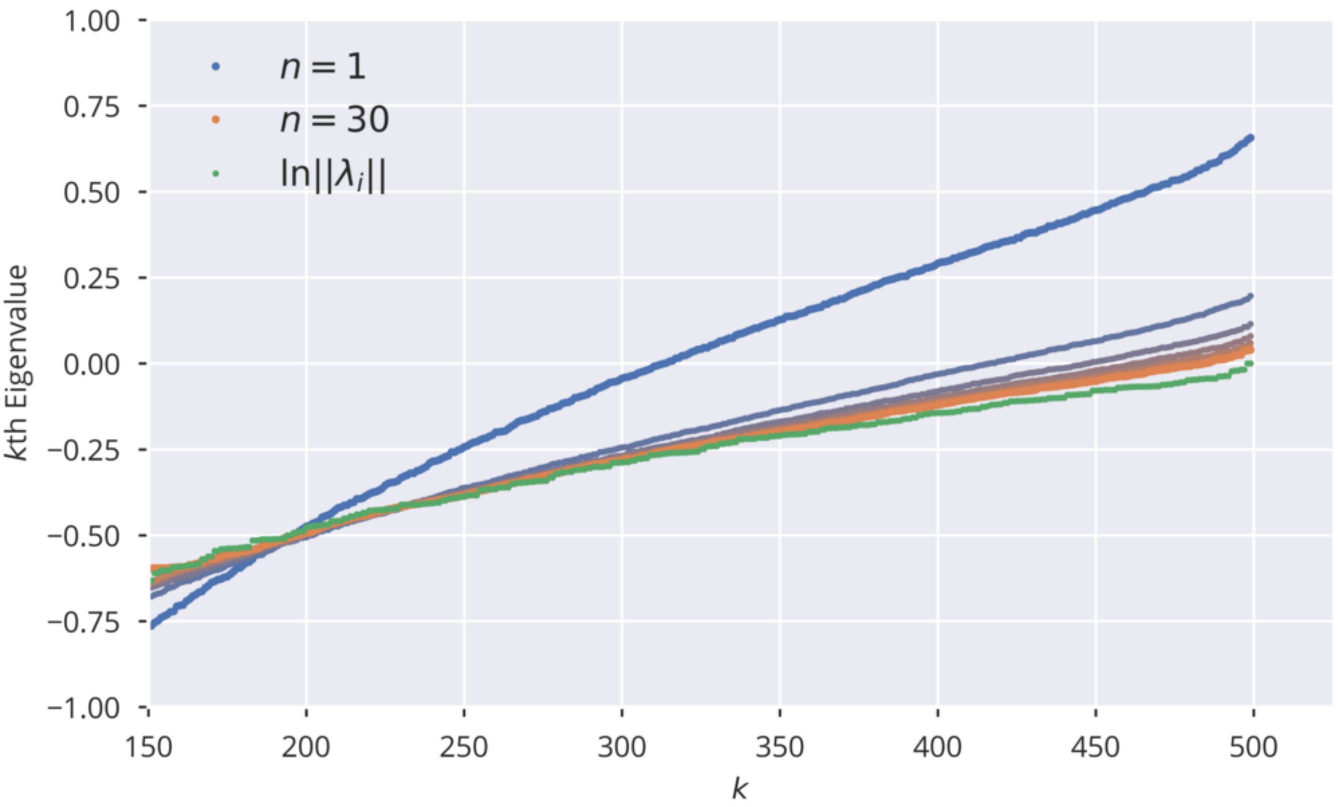

Figure S7. Convergence of Lyapunov Spectrum. Convergence of eigenvalues of $\ln\left((\widehat{W}^n)^\dagger \widehat{W}^n\right)/(2n)$ for different n, as discussed in Section 2.6. $\widehat{W}$ is a random Gaussian matrix which was rescaled to a spectral radius of one. Colors from blue to orange encode the exponent n ranging between 1 and 30. Green dots show the theoretical limit of $\ln\|\lambda_i\|$, where λ_i is the ith eigenvalue of $\widehat{W}$.

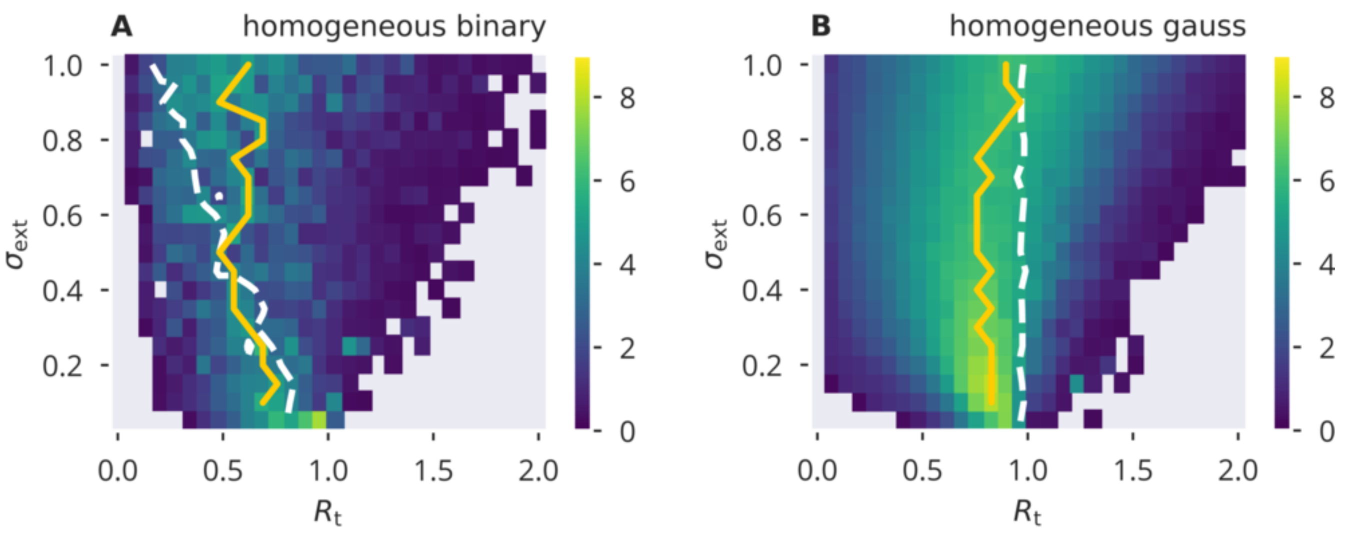

Figure S8. XOR performance for flow control, homogeneous input. Numerical results for the network performance under a time-delayed XOR task, as defined in Section 2.7, using homogeneous binary/Gaussian input. Shown are color-coded performance sweeps for the XOR-performance (18), averaged over five trials. The input has variance σ^2_{ext} and the target for the spectral radius R_{t}. A/B panels are for binary/Gaussian input protocols. Optimal performance for a given σ_{ext} is given by yellow solid lines, measured value of $R_{\mathrm{a}} = 1$ by white dashed lines.

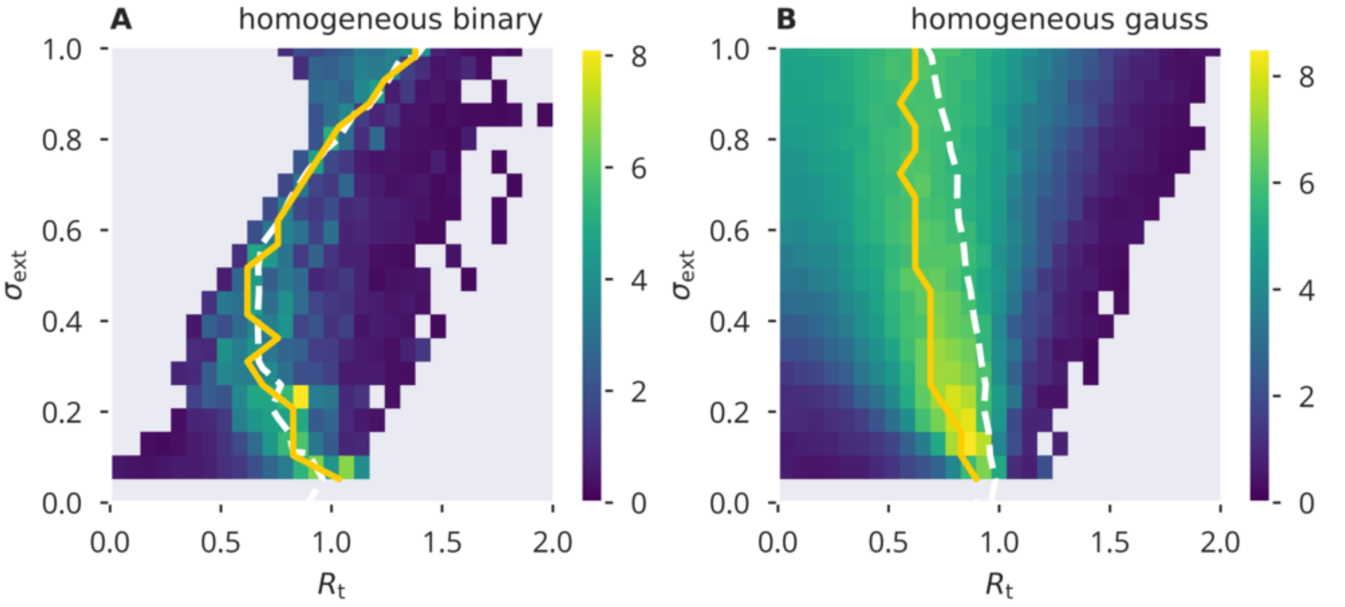

Figure S9. XOR performance for variance control, homogeneous input. Numerical results for the network performance under a time-delayed XOR task, as defined in Section 2.7, using homogeneous binary/Gaussian input. Shown are color-coded performance sweeps for the XOR-performance (18), averaged over five trials. The input has variance σ^2_{ext} and the target for the spectral radius R_{t}. A/B panels are for binary/Gaussian input protocols. Optimal performance for a given σ_{ext} is given by yellow solid lines, measured value of $R_{\mathrm{a}} = 1$ by white dashed lines.

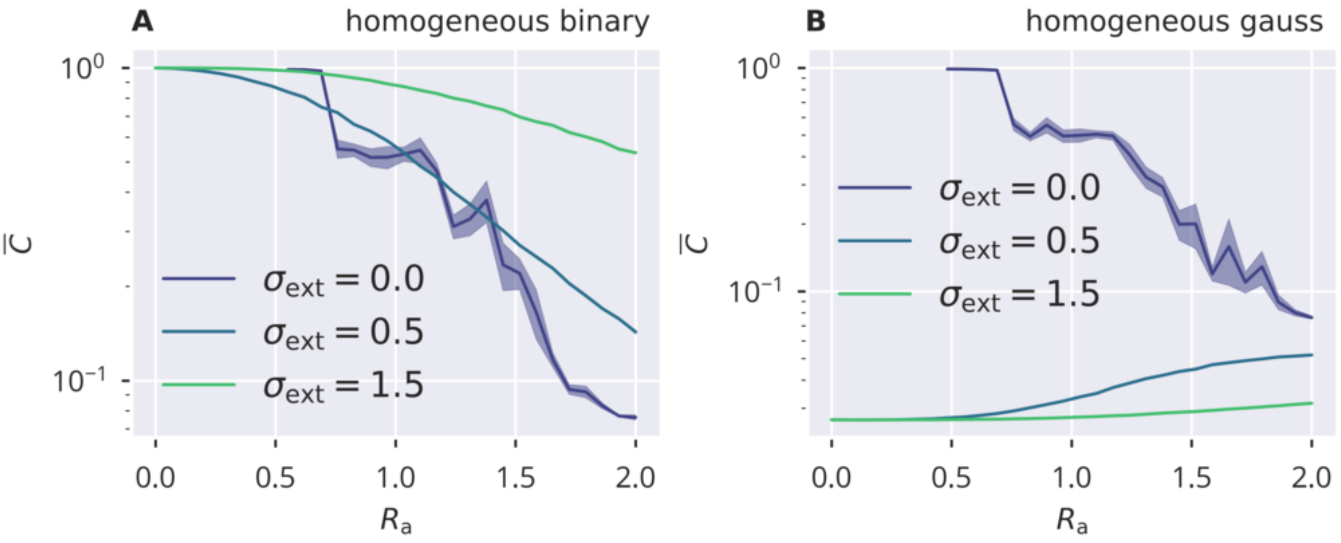

Figure S10. Input induced activity correlations. For homogeneous binary and Gaussian inputs (A/B), the dependency of mean activity cross correlations $\bar{C}$, see Eq. (20). $\bar{C}$ is shown as a function of the target spectral radius R_a. Results are obtained for $N = 500$ sites by averaging over five trials, with shadows indicating the accuracy. Correlations are due to finite-size effect for the autonomous case $\sigma_{ext} = 0$.

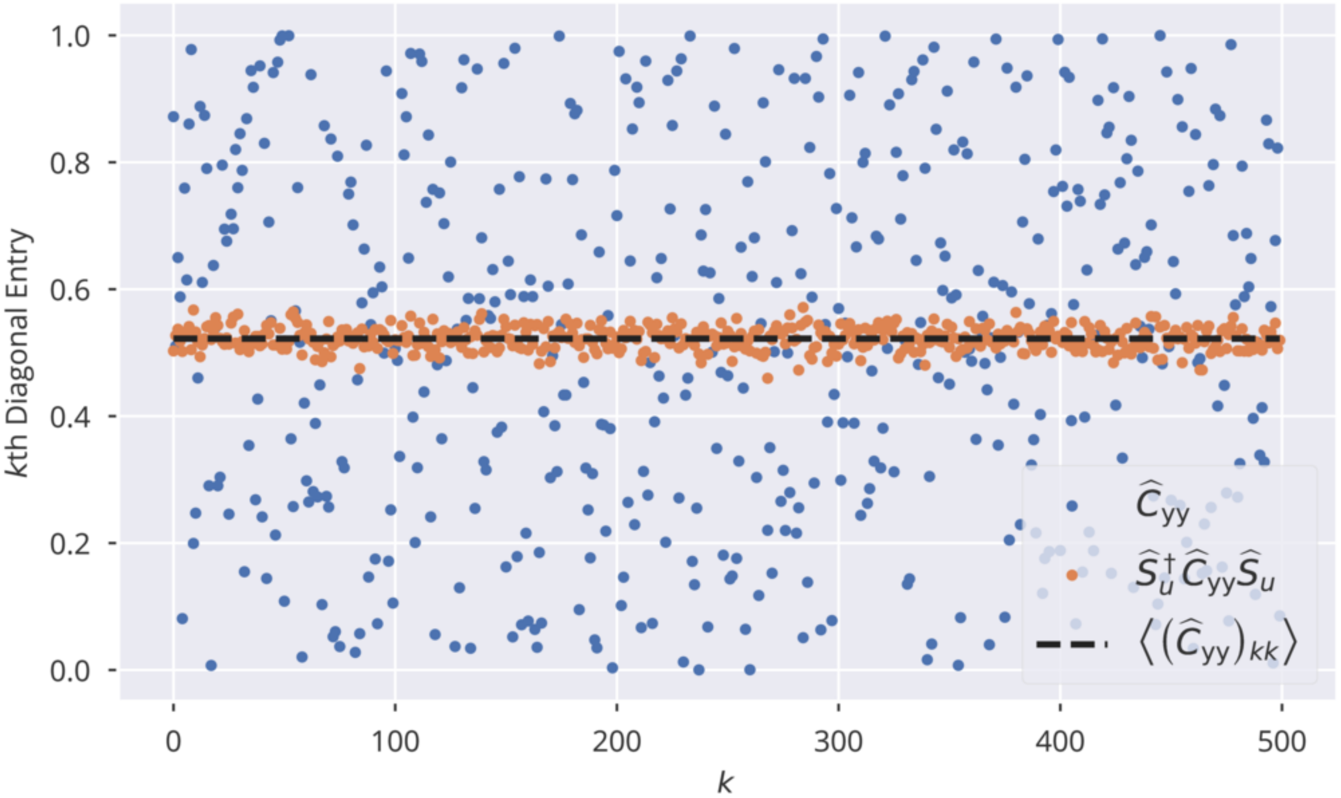

Figure S11. Diagonal Elements of a randomly generated covariance matrix and its representation in the $\widehat{\mathrm{S}}_{\mathrm{u}}$ basis. $\widehat{C}_{\mathrm{yy}}$ is a diagonal matrix with diagonal entries randomly drawn from $[0, 1]$, $\widehat{S}_{\mathrm{u}}$ is the orthonormal eigenbasis of $\widehat{W}_{\mathrm{a}}^\dagger \widehat{W}_{\mathrm{a}}$, where $\widehat{W}_{\mathrm{a}}$ is a random Gaussian matrix. The black dashed line denotes the average over the diagonal entries of $\widehat{C}_{\mathrm{yy}}$.
